# *Plasmodium falciparum* PfSET7: enzymatic characterization and cellular localization of a novel protein methyltransferase in sporozoite, liver and erythrocytic stage parasites

**DOI:** 10.1038/srep21802

**Published:** 2016-02-23

**Authors:** Patty B. Chen, Shuai Ding, Gigliola Zanghì, Valérie Soulard, Peter A. DiMaggio, Matthew J. Fuchter, Salah Mecheri, Dominique Mazier, Artur Scherf, Nicholas A. Malmquist

**Affiliations:** 1 Unité Biologie des Interactions Hôte-Parasite, Département de Parasites et Insectes Vecteurs, Institut Pasteur, Paris 75015, France; 2 CNRS, ERL 9195, Paris 75015, France; 3 INSERM, UMR 1201, Paris 75015, France; 4 Sorbonne Universités, UPMC Univ Paris 06, INSERM U1135, CNRS ERL 8255, Centre d’Immunologie et des Maladies Infectieuses (CIMI-Paris), 91 Bd de l’hôpital, 75013, Paris, France; 5 AP HP, Centre Hospitalo-Universitaire Pitié-Salpêtrière, 75013 Paris, France; 6 Department of Chemical Engineering, Imperial College London, South Kensington Campus, London SW7 2AZ, United Kingdom; 7 Department of Chemistry, Imperial College London, South Kensington Campus, London SW7 2AZ, United Kingdom

## Abstract

Epigenetic control via reversible histone methylation regulates transcriptional activation throughout the malaria parasite genome, controls the repression of multi-copy virulence gene families and determines sexual stage commitment. *Plasmodium falciparum* encodes ten predicted SET domain-containing protein methyltransferases, six of which have been shown to be refractory to knock-out in blood stage parasites. We have expressed and purified the first recombinant malaria methyltransferase in sufficient quantities to perform a full enzymatic characterization and reveal the ill-defined PfSET7 is an AdoMet-dependent histone H3 lysine methyltransferase with highest activity towards lysines 4 and 9. Steady-state kinetics of the PfSET7 enzyme are similar to previously characterized histone methyltransferase enzymes from other organisms, however, PfSET7 displays specific protein substrate preference towards nucleosomes with pre-existing histone H3 lysine 14 acetylation. Interestingly, PfSET7 localizes to distinct cytoplasmic foci adjacent to the nucleus in erythrocytic and liver stage parasites, and throughout the cytoplasm in salivary gland sporozoites. Characterized recombinant PfSET7 now allows for target based inhibitor discovery. Specific PfSET7 inhibitors can aid in further investigating the biological role of this specific methyltransferase in transmission, hepatic and blood stage parasites, and may ultimately lead to the development of suitable antimalarial drug candidates against this novel class of essential parasite enzymes.

While global mortality due to malaria has decreased since the beginning of this century, this parasitic disease continues to claim approximately 0.6 million lives per year, particularly in the vulnerable populations of children under five years of age and pregnant women^1^. Malaria eradication efforts have been hindered by the incredible ability of the parasite to develop resistance to existing antimalarials, prompting the search for novel essential factors to serve as potential drug targets. The complex life cycle of human malaria parasites involves an insect vector phase, a liver stage at the onset of infection, an asexual blood stage responsible for disease pathogenesis and a sexual stage permitting disease transmission. Transition through the various stages of the complex parasite life cycle is a highly controlled process regulated at the level of transcriptional gene activation. Indeed, in the experimentally tractable asexual blood stage, a clear stage-specific transcriptional cascade as been observed^2^. Aside from the recent discovery of a plant-derived family of transcription factors^3^, no canonical gene regulatory elements have been identified in *P. falciparum*. However, a variety of distinct epigenetic mechanisms have been discovered which contribute to the transcriptional control of single copy genes as well as the orchestration of clonally variant virulence gene families^4^,^5^,^6^,^7^,^8^.

Histone post-translational modifications (PTMs) play a particularly important role in the developmental progression of blood stage malaria parasites. The role of histone PTMs was first demonstrated in the monoallelic expression of a virulence gene family known as the *var* genes, which are involved in antigenic variation and pathogenesis^4^. Subsequent studies have shown that transcriptional activation and silencing of virtually all genes is associated with histone methylation or acetylation^5^,^6^. The tri-methylation of histone H3 lysine 4 (H3K4me3) and acetylation of histone H3 lysine 9 (H3K9ac), associated with transcriptional activation in the conserved histone code, is indeed associated with actively transcribed genes, including the single expressed *var* gene, in *P. falciparum*^5^,^6^. Tri-methylation of histone H3 lysine 9 (H3K9me3), a repressive mark in the conserved histone code, stands out in malaria parasites for its association with the variegated gene expression of clonally variant gene families and genes encoding a parasite-induced erythrocyte permeation pathway and with regulating the commitment to transmission stage parasites^5^,^6^,^9^,^10^. Importantly, H3K9me3 is not associated with general transcriptional repression across the genome^5^,^6^. Tri-methylation of histone H3 lysine 36 (H3K36), an activation mark in the conserved histone code, plays a dual function in *P. falciparum*, as H3K36me3 is associated with transcriptionally active genes throughout the genome but also is associated with silenced members of *var* genes and other clonally variant gene families^11^. Additional methylation PTMs exist on *P. falciparum* histones, some of which are conserved throughout eukaryotes while others are unique to *Plasmodium*^12^. These methylation marks exist in combination with other PTMs such as acetylation and phosphorylation^13^, but their functions, either singly or in combination, remain to be elucidated.

Protein methyltransferase enzymes (PMTs) catalyze the mono-, di- or tri-methylation of lysine residues (PKMTs) or the mono- or di-methylation of arginine residues (PRMTs). PKMTs and PRMTs were initially thought to be specific for the methylation of a single protein substrate, and while some members of this class of enzyme do exhibit a high degree of protein substrate specificity, it is widely recognized that certain individual PMTs are able to methylate multiple protein substrates, including both histones in the nucleus and non-histone proteins in the cytosol^14^. Since both PKMTs and PRMTs have been associated with a variety of diseases including cancer, neurodegenerative and inflammatory diseases, PMT enzymes have emerged as a target class for drug discovery against human disease^15^.

Recently, we have expanded our fundamental research on the role of histone PTMs in malaria parasite gene regulation to include translational research into discovering PKMT inhibitors for both the further study of parasite biology using a chemical biology approach and for the potential development of novel classes of future antimalarials. In the absence of recombinant target parasite enzymes we initiated an approach based on targeting malaria parasite histone methylation using a known inhibitor of a human PKMT, BIX-01294^16^,^17^,^18^. These studies established histone methylation to be a viable target for antimalarial drug discovery, as BIX-01294 and its derivatives cause rapid and specific parasite death with the concomitant reduction in parasite histone methylation levels^16^. These inhibitor-based results have motivated increased efforts into recombinant PfPKMT enzyme production to both fully characterize these enzymes and to enable a target-based approach to discovering specific malaria parasite enzyme inhibitors.

Computational analysis predicts ten PKMTs and three PRMTs in the *P. falciparum* genome. All ten identified PfPKMT genes contain a catalytic methyltransferase SET domain, named after the *Drosophila* chromatin-modifying enzymes Su(var)3–9, Enhancer of zeste, and Trithorax^19^. SET domain containing proteins were initially studied in the context of specifically methylating lysine residues of histones, but subsequent work has revealed numerous non-histone protein substrates for SET domain proteins^20^. Knock-out studies have indicated a subset of PfPKMTs are essential in blood stage parasites^11^. Despite significant efforts, the molecular characterization of PfPKMT enzyme activity has been constrained by the inability to produce sufficient quantities of recombinant proteins. Cui *et al.* were able to express four PfPKMTs (PfSET1, PfSET2, PfSET3, PfSET8) using a wheat germ expression system, but only assign histone H4 lysine 20 (H4K20) methyltransferase activity to PfSET8 and histone H3 methyltransferase activity to PfSET2 through Western blots analysis and autoradiography of enzyme reactions containing nucleosomes as protein substrates^21^. PfSET2, since re-named PfSETvs, was confirmed to have H3K36 methytransferase activity through the observed reduction of this mark along *var* genes in PfSETvs knock-out parasites^11^. Volz, *et al.* were able to detect low-level H3K4 methyltransferase activity for an affinity-tagged version of endogenous PfSET10 immunoprecipitated from transgenic parasites^22^. To date, no isolated PfPKMT enzymes have undergone biochemical characterization to determine substrate specificity and enzyme kinetic parameters.

In this report we describe the first large-scale production and enzymatic characterization of a recombinant *P. falciparum* PKMT, the poorly understood PfSET7, purified from a baculovirus expression system. Recombinant PfSET7 displays comparable kinetics to other characterized PKMTs from human and mouse with regards to enzyme turnover and AdoMet methyl-donor utilization. Nucleosome labeling experiments reveal that PfSET7 extensively methylates H3K4 and H3K9, but modifies the latter particularly in the presence of pre-existing acetylated histone H3 lysine 14 (H3K14ac). Immunofluorescence imaging of blood stage parasites, however, reveals PfSET7 to localize to distinct foci outside of the parasite nucleus. PfSET7 was also identified by immunofluorescence to be present in motile salivary gland sporozoite stage parasites and in liver stage parasite forms. Since PfSET7 was refractory to genetic knock-out in blood-stage parasites^11^, this enzyme is presumed to be essential in at least that stage, and therefore represents a promising antimalarial drug target candidate. With a biochemically characterized enzyme amenable to large-scale expression and purification, target based inhibitor discovery can proceed. Specific PfSET7 inhibitors will be useful tools to further explore the biological function of PfSET7 and have potential to be developed into a novel class of future antimalarials.

## Results

### Expression and purification of recombinant wild-type and mutant PfSET7

**Figure 1:**
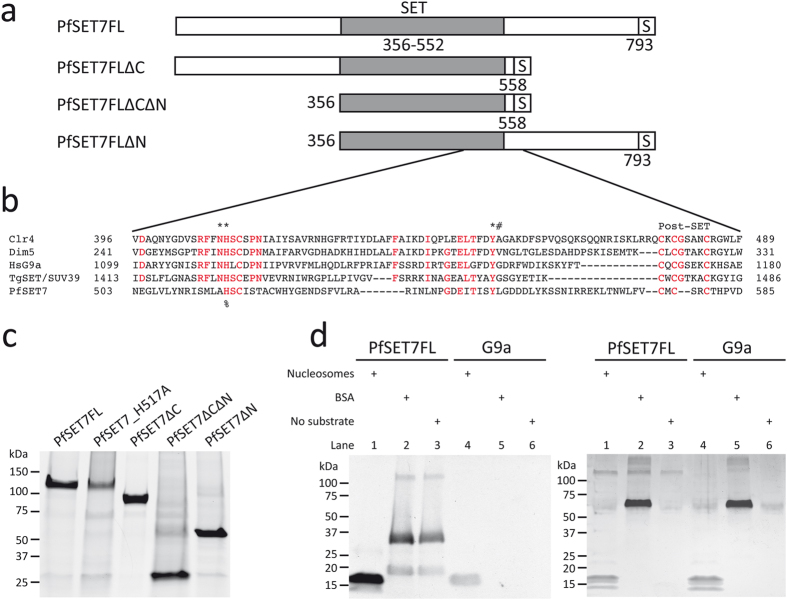
Protein production and purification. (**a**) Schematic of the PfSET7 full-length protein and truncated proteins, all expressed as recombinant affinity-tagged proteins. SET, SET domain; S, 2× strep-tag. (**b**) Alignment of the C- terminal part of the SET domain and post-SET domains of H3K9 methyltranserases from *Schizosaccharomyces pombe* Clr4 (O60016), *Neurospora crassa* Dim5 (Q8X225), *Homo sapiens* HsG9a (Q96KQ7), *Toxoplasma gondii* SET/SUV39 (TGME49_255970), *Plasmodium falciparum* PfSET7 (PF3D7_1115200). Conserved residues are highlighted in red. (*) Residues involved in catalysis. (#) Final amino acid of the SET domain. (%) Indicates residue mutated in the catalytic mutant PfSET7_H517A. Post-SET, post-SET domain. (**c**) SDS-PAGE of recombinant PfSET7FL, the catalytic mutant PfSET7_H517A, PfSET7ΔC, PfSET7ΔCΔN and PfSET7ΔN. (**d**) Autoradiograph (left panel) of enzyme reactions with PfSET7FL (lanes 1, 2 and 3) and MmG9a (lanes 4, 5 and 6) with nucleosomes, BSA and enzyme alone. Film was exposed 72h. Duplicate reactions were resolved by SDS-PAGE and silver stained (right panel).

The 793 amino acid PfSET7 protein contains a central catalytic SET domain followed by a short post-SET domain, which is flanked by a 355 amino acid N-terminal arm and 240 amino acid C-terminal arm (Fig. 1a). The SET domain is identifiable due to its homology with PKMTs from other species^21^. The C-terminal region of the PfSET7 SET domain (Fig. 1b), which contains the catalytic residues, shows higher homology to other organisms compared to the N-terminal region^21^. As with most of the *Plasmodium* genome, the PfSET7 gene contains a high percentage (72%) of AT nucleotides. To reduce the high AT content for heterologous recombinant protein expression we obtained a codon optimized version of wild-type full-length PfSET7 (PfSET7FL). In anticipation of potential recombinant protein production in secretory systems, two potential N-glycosylation sites were mutated. Recombinant protein expression attempts in mammalian cells and wheat germ were unsuccessful. Ultimately, an active recombinant PfSET7 enzyme fused to a C-terminal 2x-strep tag was successfully produced in a baculovirus-based Sf9 insect cell expression system.

To study the impact of the N- and C-termini on methyltransferase activity, truncated mutants of PfSET7 (Fig. 1a) were made by removing the N-terminus or the C-terminus or both termini, leaving only the SET domain. Finally, to confirm that our purified methyltransferase activity is specific to PfSET7, a catalytically inactive mutant, PfSET7_H517A, was produced by the mutation of a single conserved catalytic residue.

Purified PfSET7FL and PfSET7_H517A are expected to migrate at 98 kDa, though a single protein band is consistently observed at approximately 120 kDa (Fig. 1c). PfSET7ΔC is expected at 70 kDa but migrates at 85 kDa. PfSET7ΔCΔN and PfSET7ΔN are both expressed at an expected size of 27 and 55 kDa, respectively. Recombinant PfSET7 proteins nonetheless were purified to homogeneity.

### Steady state enzyme kinetics

To investigate the methyltransferase activity of PfSET7FL, the purified enzyme was tested in a PKMT assay using methyl-^3^H-AdoMet as the methyl donor. Mouse G9a, a known histone H3K9 PKMT was used as a positive control. Both enzymes were tested for methyltransferase activity in the presence of nucleosomes as a protein substrate, with BSA as a general protein substrate, or without a protein substrate. Enzyme reaction contents were migrated on SDS-PAGE and exposed to film (Fig. 1d left panel). A second gel loaded with identical reactions using unlabelled AdoMet was silver stained to show total protein content (Fig. 1d right panel). Methyltransferase activity of PfSET7FL was confirmed by the presence of bands on the autoradiograph. In reactions with PfSET7FL and G9a with nucleosomes (Lanes 1 & 4), a band at 17 kDa corresponding to histone H3 is apparent. PfSET7FL produces a second band below H3, corresponding to histone H4. There was no methyl transfer to BSA by either enzyme (lanes 2 & 5). However, in the absence of nucleosomes or in the presence of BSA, PfSET7FL transfers methyl groups to proteins that migrate at 19 kDa, 32 kDa and 125 kDa (Lanes 2 & 3). The band at 125 kDa co-migrates with PfSET7FL and could represent automethyation of the enzyme occurring in the absence of nucleosomes, as these bands are absent when nucleosomes are present^23^. The two lower bands could be methylation of degradation fragments of the full-length enzyme, as there are no other common proteins added to these reactions. These results suggest PfSET7FL is primarily a histone H3 methyltransferase, with possible additional activity toward histone H4.

**Figure 2:**
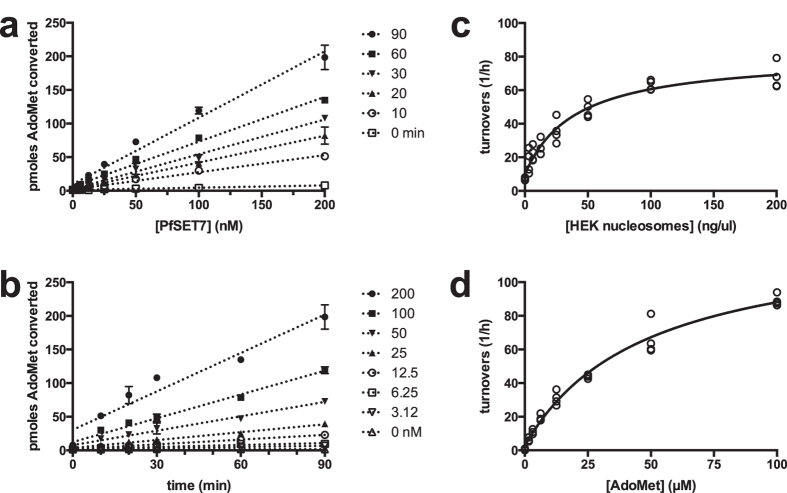
Steady state enzyme kinetics of PfSET7FL. (**a**) Enzyme concentration and (**b**) time dependent enzyme activity. (**c**) Saturation kinetics for HEK nucleosomes yield a Km value of 40 ± 9 ng/ul. (**d**) Saturation kinetics for methyl-donor AdoMet yield a Km value of 48 ± 7 μM. Data in (**a**,**b**) are mean ± SD of one representative experiment. Data in (**c**,**d**) are replicate values from duplicate experiments and were fitted to the Michaelis-Menton equation using GraphPad Prism software.

To characterize the enzymatic properties of PfSET7FL, time and enzyme concentration dependent activity of PfSET7FL was examined using radiometric assays (Fig. 2a,b). Enzyme activity was linear from 12.5–200 nM for up to 90 minutes of reaction time. Subsequent experiments therefore used 25 nM enzyme in 60 minutes reactions to remain in this linear range. Nucleosome-dependent activity was tested at nucleosome concentrations of 0–200 ng/ul, revealing a Km 40 ± 9 ng/ul (Fig. 2c). AdoMet-dependent activity was examined at AdoMet concentrations of 0–100 μM, yielding a Km for AdoMet of 48 ± 7 μM (Fig. 2d). Subsequent experiments used nucleosome and AdoMet at saturating concentrations of 200 ng/ul and 100 μM, respectively. PfSET7FL revealed a turnover of between 80 to 150 h^−1^, which is comparable to other characterized PKMTs such as mouse G9a at 88 h^−1^ 24 and Dim5 at 180 h^−1 ^25.

**Figure 3:**
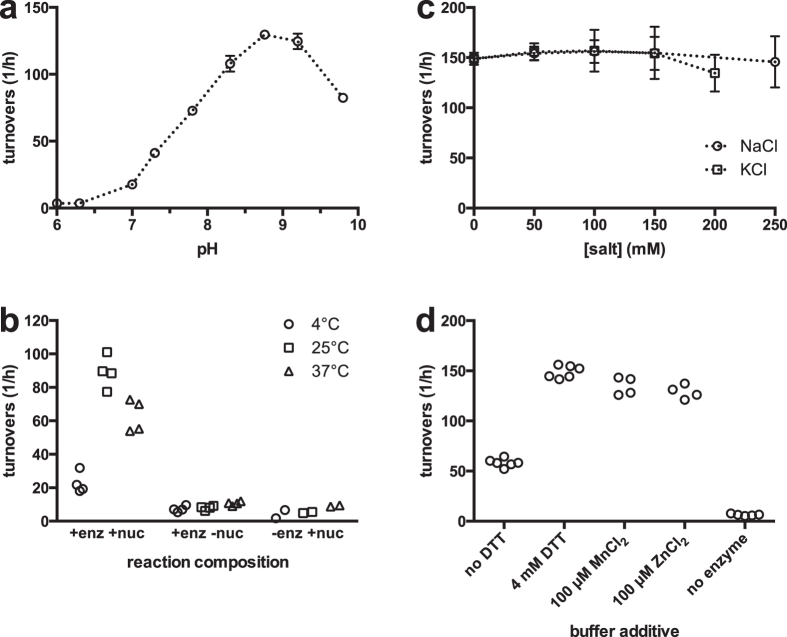
Characterization of PfSET7FL enzyme reaction conditions. (**a**) PKMT reactions were performed to determine pH dependent enzyme activity on a pH range of 6–10. (**b**) Temperature dependent enzyme activity where reactions were carried out at 4 °C (circles), 25 °C (squares), or 37 °C (triangles). Control reactions without nucleosomes or without enzyme were performed in parallel. (**c**) The effect of two common salts NaCl (circles) and KCl (squares) were tested up to 250 mM or 200 mM, respectively. (**d**) The effect of removing DTT or adding divalent metal ions on enzyme activity. All reactions, except where noted, contain 4 mM DTT. Reactions contained 100 μM MnCl_2_ or ZnCl_2_ where noted. Data from at least two independent experiments are represented in (**a**,**c**) as mean ± SD. Individual data from two independent experiments are presented in (**b**,**d**).

Experiments to test pH dependence showed PfSET7FL to display peak activity near pH 8.8 (Fig. 3a), with activity decreasing above and below this pH value. Performing enzyme reactions at three different temperatures of 4 °C, 25 °C and 37 °C showed the highest activity at 25 °C (Fig. 3b). The presence of monovalent salts has been reported to be detrimental to PKMT activity for certain recombinant enzymes. However, up to 250 mM NaCl or 200 mM KCl (Fig. 3c) does not appear to affect PfSET7FL enzyme activity. Various PKMT characterization buffers reported in the literature contain divalent magnesium cations. To investigate any role for magnesium on PfSET7FL, MgCl_2_ was added at 100 μM but did not appear to alter PfSET7FL enzyme activity. SET domain proteins are known to contain zinc binding sites^26^, thus the effect of zinc ions on PfSET7FL enzyme activity was tested. The data show supplementing reactions with 100 μM zinc has little effect on apparent enzyme activity. Finally, the presence of the reducing agent dithiothreitol (DTT) was tested, and the data reveal 4 mM DDT results in increased apparent PfSET7 enzyme catalysis relative to no reducing agent present (Fig. 3d). These data define the optimal assay conditions for PfSET7FL for the present analyses for any future *in vitro* PfSET7FL studies, including further enzyme characterization or subsequent inhibitor discovery.

### PfSET7 mutational analysis

**Figure 4:**
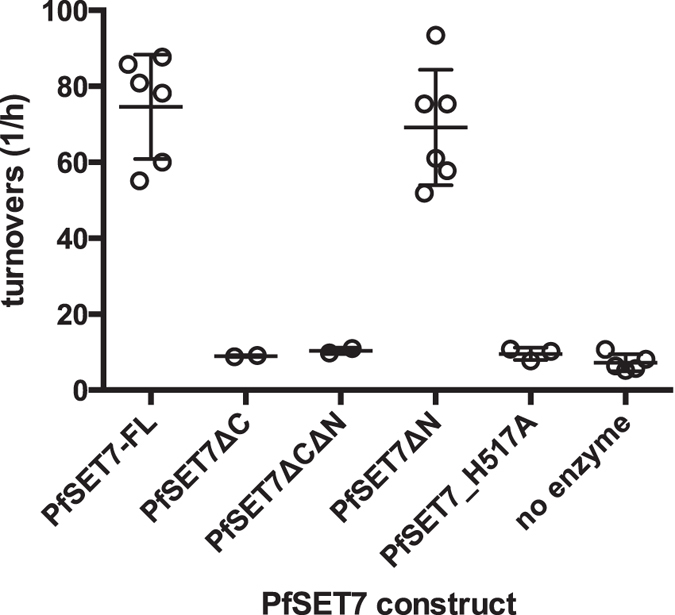
PfSET7 mutational analysis. Enzyme activity of full-length, truncated and catalytic dead versions of PfSET7. Data from two independent experiments are represented as mean ± SD.

To identify functionally important regions of PfSET7, truncation mutants were examined for catalytic activity (Fig. 4). PfSET7ΔN demonstrated PKMT activity similar to that of the full-length enzyme, indicating that the N-terminus of PfSET7 is not essential for enzyme activity.

While some PKMT enzymes contain only a catalytic SET domain^27^, others also possess a cysteine rich post-SET domain that is implicated in protein folding and formation of the active site^28^. In proteins containing a post-SET domain, this domain has been shown to be necessary for enzyme activity^29^. PfSET7 contains a cysteine rich putative post-SET domain similar to post-SET domains from other PKMTs, though the canonical CxCxxxxC motif as seen in Dim5 and G9a is a CxCxxC motif in PfSET7 (Fig. 1b). To test if the putative post-SET domain of PfSET7 is essential for enzyme activity, two mutants lacking the post-SET domain, PfSET7ΔC and PfSET7ΔCΔN, were produced. These mutants display no enzyme activity, indicating that the putative post-SET domain of PfSET7 may indeed be necessary for catalysis as seen in other PKMT enzymes containing post-SET domains.

Wild-type PfSET7 has two conserved catalytic residues in the SET domain (Fig. 1b), H517 and Y551^21^. A third catalytic residue, N516, is present in PKMTs such as Clr4, Dim5 and HsG9a, but absent in PfSET7, which contains an arginine at this location. Mutation of the conserved H517 has been shown to abolish PKMT activity^30^,^31^. Accordingly, the catalytic mutant PfSET7_H517A shows no HKMT activity compared to PfSET7FL (Fig. 4). This confirms that the methyltransferase activity we observe is specific to PfSET7 and that mutation of a single conserved residue is sufficient to abolish the activity of this purified recombinant enzyme.

### Mass spectrometry identification of PfSET7 histone lysine methylation targets

To identify the target amino acid specificity of PfSET7, we employed deuterium-labeled AdoMet (CD3-AdoMet) and isolated human nucleosomes in a mass spectrometry based enzyme assay. These experiments have the advantage of providing a protein substrate containing a range of pre-existing histone PTMs and can identify an enzyme preference for specific target residues and any influence of modifications to neighboring residues. Recombinant PfSET7 enzyme (1 μM) was incubated with human nucleosomes (0.1 mg/mL, isolated from HEK293 nuclei) and CD3-AdoMet (1 mM) for 3 hours. Histones were prepared for mass spectrometry analysis (as described in the Methods section) to identify the lysine targets that were methylated by PfSET7, which are distinguishable from existing methylations by a CD3 mass shift (+3 Daltons) in the m/z of the modified peptide. A mass shift of 3 Daltons is necessary to sufficiently distinguish new methyl groups added by the recombinant HKMT from previously existing histone methylation, as naturally occurring isotopes (e.g. ^13^C, ^15^N, etc) and their combinations result in abundant isotopic peaks that are +1 and +2 Daltons after the monoisotopic peak (i.e. the mass based on the most abundant isotopes: ^12^C, ^14^N, ^16^O, etc). The same *in vitro* labeling experiment was performed using recombinant mouse G9a as a reference control as it is known to methylate H3K9. For instance, the right panels of Figures S1 and S2 reveal significant levels of mouse G9a methylation for H3K9me3K14ac and H3K9me2K14un, respectively, where the isotopic peak cluster for every methylation event has been colour-coded based on the total number of CD3 methyl groups (blue, green and red isotopic peaks represent the signal for 1, 2 and 3 CD3 methyl groups, respectively).

**Figure 5:**
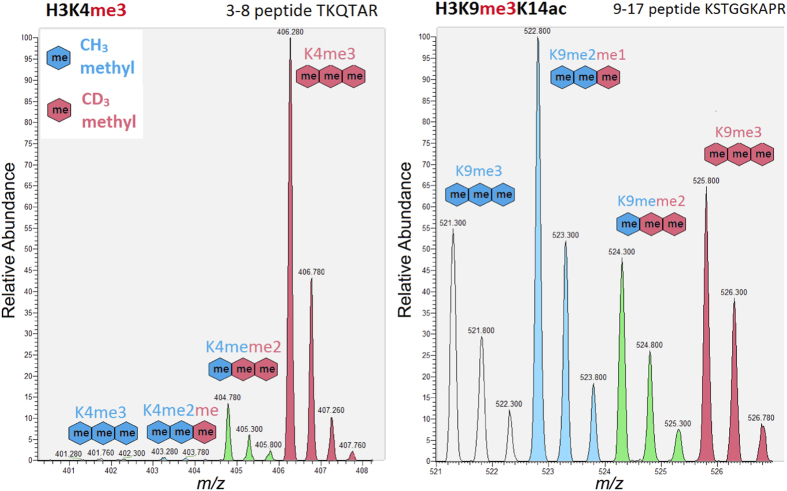
PfSET7 extensively methylates H3K4 and H3K9 in the presence of H3K14ac. Recombinant PfSET7 enzyme was incubated with human nucleosomes and CD3-labeled S-adenosyl-methionine to identify the histone lysine targets of PfSET7, which are distinguishable from existing methylations (blue colored ‘me’ symbols) by a CD3 mass shift (+3 Daltons; red colored ‘me’ symbols) in the m/z of the modified peptide. Significant labeling of H3K4me2 and H3K4me3 by PfSET7 was observed (shown for H3K4me3 in the left panel), where the major product is the fully methylated H3K4 substrate. Extensive labeling of H3K9me2 and H3K9me3 by PfSET7 was also observed, but particularly in the presence of existing H3K14 acetylation on nucleosome substrates (shown for H3K9me3K14ac in the right panel), implying this methyltransferase exhibits some degree of nucleosome specificity (compare extent of labeling of H3K9me2K14un substrate by PfSET7 and mG9a in Supplementary Fig. S2). Annotated tandem mass spectra for all observed labeled histone peptides are provided in the Supplementary Figures.

Extensive labeling of H3K4 di- and tri-methylation (H3K4me2, H3K4me3) was observed for PfSET7 (left panel of Fig. 5 for H3K4me3; H3K4me2 in Fig. S1 and corresponding tandem mass spectra in Figures S3–S7), but not for mouse G9a. Interestingly, PfSET7 appears to modify H3K4 to its highest methyl occupancy, as observed by the peak intensities in the mass spectrum (in the left panel of Fig. 5, the highest intensity peak corresponds to H3K4me3 with three new CD3 methyl groups; similar trend observed in Fig. S1 for H3K4me2). PfSET7 was also observed to methylate H3K9 in the presence of existing K14 acetylation on nucleosome substrates (right panel of Fig. 5 for H3K9me3K14ac; corresponding tandem mass spectra in Figures S8–S13) to the same extent as mouse G9a (compare Figs. 5 to S1). However, whereas mouse G9a exhibited a similar degree of H3K9 methylation activity for unmodified H3K14 substrates (right panels of Figs S1 and S2), the extent of PfSET7-mediated K9 methylation was orders of magnitude lower in the absence of H3K14 acetylation, as shown in Fig. S2 for H3K9me2K14un (see also tandem MS in Figures S14–S17). This implies PfSET7 preferentially recognizes nucleosome substrates containing acetylation on H3K14.

To a lesser extent PfSET7 was also observed to methylate H3K36, particularly when H3K27 is unmodified (see Figures S18 and S19 for H3K36me1 and H3K36me3, respectively). A tandem mass spectrum was identified to support evidence of PfSET7 methylation of H3K27me2 substrates to H3K27me3 (Fig. S20). However, it should be noted that methylation activity for H3K27me2/me3 is also observed for mouse G9a, which can occur under *in vitro* conditions since the amino acid sequences around H3K9 and H3K27 are homologous (AR**K**ST and AR**K**SA, respectively). Methylation of other common lysine targets (i.e. H3K18, H3K23, H3K79 and H4K20) was not observed for PfSET7.

### Cellular localization of PfSET7 in blood stage parasites

**Figure 6:**
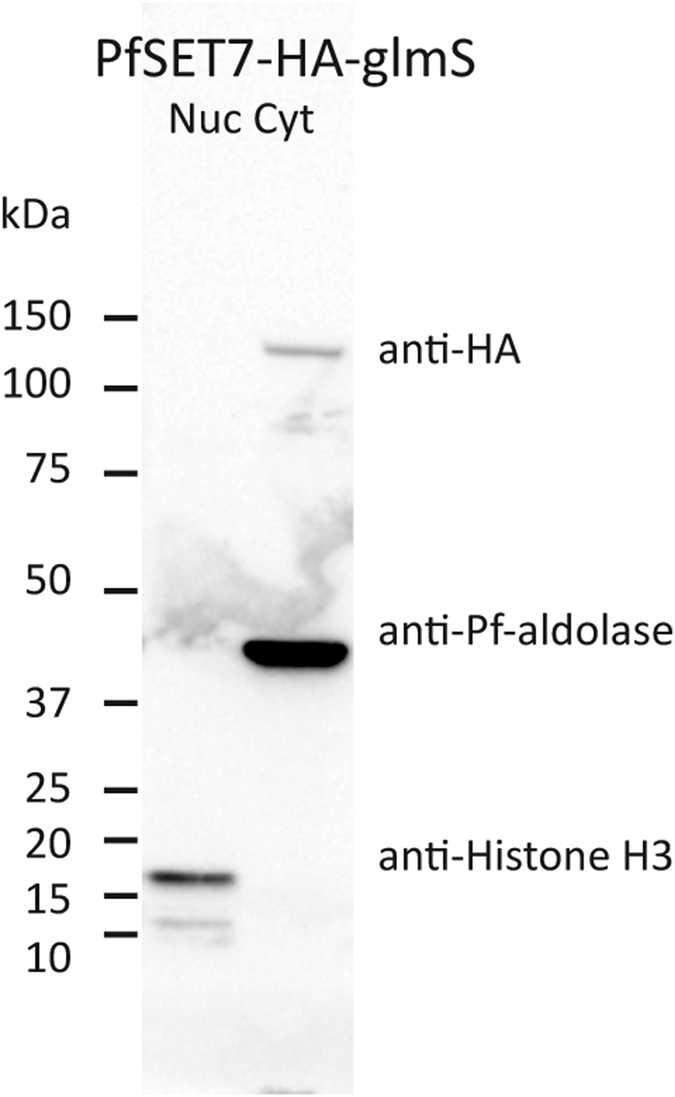
PfSET7 expression and cytoplasmic localization. Western blot of nuclear and cytoplasmic fractions of asynchronous parasites using anti-HA antibodies against PfSET7-HA-glmS. The two fractions were monitored for purity using control antibodies anti-Pf-aldolase (predicted MW: 29 kDa) for the cytoplasm and anti-histone H3 (predicted MW: 15 kDa) for the nucleus. Anti-HA recognized the PfSET7-HA band of >100 kDa (predicted MW: 98 kDa) in the cytoplasm.

To identify the cellular location of PfSET7 in parasites, a Pf3D7-SET7-HA-glmS integrant transfected line was generated through markerless insertion at the PfSET7 genomic locus of a 3 × HA affinity tag and the glmS ribozyme using the CRISPR/Cas9 genome editing tool. Correct genomic integration of this transgenic parasite line was confirmed by PCR and endogenous tagged protein expression was confirmed by Western Blot (Fig. S21). This glmS construct was designed to produce an inducible knock-down parasite line, but was found to be inefficient at PfSET7 mRNA and protein reduction in the presence of glucosamine inducer, and induced parasites displayed no obvious growth phenotype (Fig. S22). To determine the subcellular distribution of PfSET7, whole cell extracts of the integrant Pf3D7-SET7-HA-glmS line were biochemically separated into nuclear and cytoplasmic fractions, which were examined by Western Blot for PfSET7-HA via the HA-tag and for aldolase or histone H3 for cytoplasmic or nuclear protein controls, respectively. The results show PfSET7 is present in the cytoplasmic fraction but not visible in the nuclear fraction (Fig. 6). These results suggest PfSET7 may have a role outside of the nucleus, which is unexpected for an apparent histone methyltransferase.

### PfSET7 localizes to a distinct cytoplasmic compartment in blood stages

**Figure 7:**
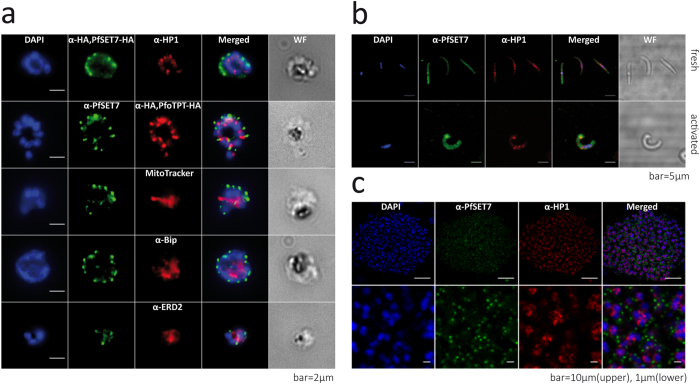
Immunofluorescence localization of PfSET7 at different stages of the *Plasmodium falciparum* life cycle. (**a**) Asexual blood stage parasites. Markers used were: DAPI as a DNA marker, anti-HA in PfSET7-HA parasites, anti-PfSET7 in wild-type parasites, anti-HP1 as a nuclear periphery marker, anti-HA in PfoTPT-HA parasites as an apicoplast marker, MitoTracker Deep Red FM staining for the mitochondrion, anti-Bip as an ER marker and anti-ERD2 as a cis-golgi marker. The scale bars correspond to 2 μm. (**b**) Mosquito salivary gland stage sporozoites. The PfSET7 is expressed in fresh (upper panel) and activated sporozoites (lower panel) characterized by the transformation bulb. Sporozoites are visualized by DAPI staining, anti-PfSET7 and anti-HP1 immunostaining. The scale bars correspond to 5 μm. (**c**) Liver stage parasites. Upper panel: liver stage schizonts from TK-NOG humanized mice were immunostained with anti-PfSET7 and anti-HP1 at day 7 post-infection. The scale bar corresponds to 10 μm. Lower panel: higher magnification of the image above. The scale bar corresponds to 1 μm.

To assess the intracellular localization of PfSET7, *P. falciparum* parasites were analyzed by immunofluorescence microscopy (IF) using monoclonal anti-HA or polyclonal anti-PfSET7 antibodies in PfSET7-HA-glmS or wild-type parasites, respectively. Staining patterns using either antibody are virtually identical, suggesting both antibodies are equivalent in detecting PfSET7, and that affinity tagging the endogenous protein does not lead to aberrant expression or localization. (Fig. S23). Through the three major asexual blood stages, PfSET7 signal exhibited a punctate pattern adjacent to the nucleus, with a concomitant increase in staining during progression through mature trophozoites and schizonts (Fig. S24). To determine whether PfSET7 is localized at the nuclear periphery or outside of the nucleus, PfSET7 staining was compared to that of PfHP1, which localizes to discrete foci within the nucleus but at the nuclear periphery^32^. Consistent with the biochemical fractionation experiments, PfSET7 staining is distinct from the nuclear periphery marker PfHP1 (Fig. 7a), further supporting PfSET7 cytoplasmic localization. The punctate fluorescent signal suggests PfSET7 could be localized to a cytoplasmic organelle rather than evenly distributed throughout the cytoplasm.

The discrete IF staining of PfSET7 led us to explore whether the protein localizes to other DNA-containing organelles in malaria parasites, namely the single parasite mitochondrion or the apicoplast, an organelle specific to the phylum apicomplexa. A previous study reported PfSET5 localizes to the mitochondria, but did not examine PfSET7^33^. To determine if PfSET7 is in the parasite mitochondrion, we combined IF staining for PfSET7 and MitoTracker Deep Red FM staining for the mitochondrion. The data show PfSET7 does not co-localize with mitochondrial staining and is therefore not located within this organelle (Fig. 7a). To determine if PfSET7 is in the apicoplast we used a parasite line expressing HA-tagged triosephosphate transporter (PfoTPT-HA), a polytopic membrane protein of the apicoplast^34^. The IF images show no signal overlap between PfSET7 and PfoTPT, indicating PfSET7 is not located in the parasite apicoplast (Fig. 7a). Other markers for organelles whose ontogeny begins in early stage were used to evaluate their proximity to PfSET7, including anti-Bip for the endoplasmic reticulum and anti-EDR2 for the cis-golgi apparatus (Fig. 7a). Results from IF studies utilizing markers for these two organelles reveal that PfSET7 does not co-localize with either the endoplasmic reticulum or the cis-golgi. These combined IF results suggest PfSET7 is localized to a distinct compartment in the cytoplasm.

### PfSET7 expression in *P. falciparum* sporozoites and in liver stage schizonts

To characterize PfSET7 expression in pre-erythrocytic parasite stages, immunofluorescence imaging was performed on *P. falciparum* mosquito salivary gland stage sporozoites and on hepatic forms. As motile sporozoites productively invade hepatocytes, these forms undergo a radical morphological remodeling, a process first discernible by a bulbous expansion that progressively enlarges to become the early spherical hepatic stage^35^. PfSET7 staining of freshly isolated sporozoites shows an uneven and clustered localization in the cytoplasm (Fig. 7b). Upon sporozoite transformation into early hepatic forms under axenic conditions, a similar cytoplasmic staining is observed with a more intense focal signal at the growing bulb (Fig. 7b). Nearly fully mature hepatic schizonts (just prior to merozoite egress) were imaged in liver sections from humanized mice seven days post-sporozoite inoculation (Fig. 7c). PfSET7 is found in these hepatic schizonts in a punctate pattern as distinct dots observed near the nucleus of each forming merozoite. This cytoplasmic and nucleus-associated fluorescence pattern is similar to that observed in asexual blood stage parasites.

## Discussion

Epigenetic gene regulation plays a critical role in malaria parasites in the control of general transcriptional regulation, monoallelic expression of virulence genes and in the commitment to transmission stage development. A major mechanism of epigenetic regulation in *P. falciparum* is mediated by histone post-translational modifications, which have been linked to transcriptional activation throughout the *Plasmodium* genome and to transcriptional repression of *P. falciparum* multi-copy gene families^36^. Building upon our fundamental research into parasite gene regulation, we have taken a chemical biology approach to target malaria methyltransferase enzymes in an effort to identify the biological role of individual PfPKMTs, ideally through the development of specific enzyme inhibitors. We have recently reported the identification and development of one PKMT inhibitor series entirely through phenotypic readouts^16^,^17^,^18^, but the inability to produce functional recombinant PfPKMTs has greatly limited our ability to take a target-based approach to PfPKMT inhibitor discovery. Indeed, the difficulty with producing recombinant PfPKMT enzymes by us and other researchers has greatly limited the functional understanding of this entire class of parasite enzymes. Notably, we tested numerous bacterial, eukaryotic and *in vitro* protein expression systems before successfully producing sufficient quantities of active PfSET7 enzyme using a baculovirus-based insect cell expression system.

Here we report the first large-scale production of an active full-length recombinant PfPKMT, allowing a detailed *in vitro* enzyme kinetics investigation of this important enzyme class in malaria parasites. PfSET7 exhibits kinetic characteristics similar to other PKMTs from other organisms in terms of steady-state AdoMet cofactor utilization, peak activity at pH ~9 and a dependence upon a catalytic histidine residue^24^,^27^,^37^. Mutational analysis of truncated PfSET7 enzymes revealed at least the post-SET domain of the C-terminal protein beyond the SET domain is required for PKMT activity, as truncated enzymes lacking this segment of the protein were inactive. PfSET7 does not exhibit decreased activity at increased salt concentrations, as has been reported for some PKMT enzymes when using peptide substrates but not nucleosome protein substrates, a result that has previously been suggested to support the interpretation that nucleosomes are the physiological substrate of a given PKMT^38^. Methyl transfer to isolated nucleosome protein substrates is saturable, further suggesting histones are a legitimate protein substrate for PfSET7. As additional support that PfSET7 is a histone methyltransferase, nucleosome labeling studies using heavy-labeled AdoMet reveals that PfSET7 extensively methylates H3K4 and H3K9, the latter target particularly in the presence of a pre-existing H3K14ac mark. Despite the specific methylation of histones, which are overwhelmingly present in the parasite nucleus, Western blot analysis and immunofluorescence localization reveal PfSET7 is detected primarily in the cytosol in blood stage parasites. Additional immunofluorescence localization in salivary gland sporozoites and liver stage parasites reveals PfSET7 to be localized throughout the cell in sporozoites and, in liver stage parasites, located at distinct foci in the cytosol adjacent to parasite nuclei similar to blood stage parasites. The specific role of PKMT enzymes in sporozoites has not been studied. PKMT enzymes in liver stage hypnozoite forms of *P. cynomolgi* malaria parasites have been linked to the maintenance of quiescence^39^, though no specific PfPKMT has been implicated in this process.

The functional significance of H3K4, H3K9 and to a lesser extent H3K36 methylation by PfSET7 is not presently understood. The methylation of H3K9 in the presence of existing H3K14 acetylation has been reported previously in mass spectrometry-based analyses of *P. falciparum* histones^12^, indicating this is indeed a bona fide histone PTM combination present in malaria parasites. By ChIP analysis, the H3K9me3 mark in *P. falciparum* has been closely linked to the monoallelic expression of genes encoding variant surface antigens^5^,^6^. Importantly, these ChIP studies used commercially available antibodies specific for the single H3K9me3 histone modification, which would presumably not discriminate between the presence or absence of H3K14ac with regards to H3K9me3. Further investigation could therefore determine whether the H3K9me3K14ac mark is responsible for regulating a subset of the genes associated with H3K9me3, and thus implicate PfSET7 in an even finer level of epigenetic control over multi-copy gene families. This finding also highlights the strengths of using mass spectrometry to detect heavy methyl labeling of modified nucleosome substrates, as assays employing unmodified histone substrates (peptides, histone proteins or nucleosomes) would fail to detect such activity. While methylation of H3K4 and H3K9 (in the presence of K14ac) methylation by PfSET7 were observed to be most abundant, further studies will be required to elucidate the significance of the H3K27 and H3K36 methylations observed in lower abundance (Figs S18–S20). Notably, the control experiment using mouse G9a enzyme reveals similar methylation of H3K27me3, perhaps resulting from the sequence homology between H3K9 and H3K27, and thus could represent a consequence of the *in vitro* assay conditions. However, the PfSET7 mediated methylation of H3K36 in the context of unmethylated H3K27 substrates is not observed in the mouse G9a enzyme control, and thus may represent an additional interesting target to investigate further.

The primarily cytosolic localization of PfSET7 in asexual blood stage parasites was unanticipated for a putative histone methyltransferase. However, PKMT enzymes which are localized in both the nucleus and the cytosol have been reported, including the human histone H3K9 specific PKMT SetDB1^40^. As well, newly synthesized histones have been reported in other organisms to be methylated at H3K9 and acetylated at H3K14 within histone-chaperone protein complexes in the cytoplasm^41^,^42^. This opens the possibility that PfSET7 is a cytosolic histone methyltransferase acting on newly synthesized histones in a PfSET7-defined subcellular cytosolic compartment. Future pull-down studies could confirm whether similar histone-chaperone protein complexes exist in *Plasmodium*. Alternatively, PfSET7 may methylate recycled, pre-modified histones. Very little is known about the order of addition of histone PTMs or histone recycling in *Plasmodium*, and additional investigation of PfSET7 through genetic or chemical attenuation may be able to address the cytosolic role of PfSET7 on the methylation of newly synthesized or recycled histones. Alternatively, many PKMT enzymes which were originally reported to be histone methyltransferases were subsequently found to possess one or more additional non-histone protein substrates^15^. As such, it is entirely possible that PfSET7 has additional non-histone protein substrates in the parasite cytosol, though further studies would be required to address this hypothesis.

Altogether, the production and enzymatic characterization of recombinant PfSET7 enzyme now allows for small molecule inhibitor discovery against this histone methyltransferase previously reported to be essential in blood stage parasites^11^. Inhibitors identified through target-based discovery can be used as tools to dissect the biological role of PfSET7 with regards to histone and potential non-histone protein methylation, and the role of the H3K9me3K14ac histone PTM combination for which PfSET7 appears to be specific. Chemical biology experiments using PfSET7 specific inhibitors can be corroborated using, for example, inducible knock-down of PfSET7 in blood stage parasites, the stage in which various genetic techniques have been established and continue to be developed. However, chemical genetics investigations using specific PfSET7 inhibitors may prove much more powerful in determining the biological role of PfSET7 in sporozoite and liver stage parasites, which are much less amenable to genetic manipulation, but present fascinating biology as the obligate initial stage of malaria infection and are responsible for malaria relapses in *P. vivax* and *P. ovale* infections. Ultimately, successfully developed small molecule inhibitors of PfSET7 may enter the antimalarial drug discovery pipeline as a much needed new chemical entity against this novel target class.

## Methods

### Protein expression constructs

Codon optimized full-length PfSET7 (PfSET7FL) gene (PlasmoDB Gene ID PF3D7_1115200) was synthesized (Genscript), fused to a C-terminal double Strep-tag and ligated into the transfer vector pVL1393. Two potential glycosylation sites in PfSETFL, S122A and T784A, were mutated to allow expression in secretory systems. The SET domain of PfSET7 was identified using Prosite (prosite.expasy.org). Truncated constructs of PfSET7 were generated by PCR (Pfu Ultra II, Agilent Technologies) using specific primers (Table S1). PfSET7ΔC (residues 1–558), PfSET7ΔCΔN (residues 356–558), PfSET7ΔN (residues 356–793) and the double strep-tag, were amplified and inserted into pVL1393 via Gibson assembly (NEB). The PfSET7_H517A catalytic mutant was generated by site directed mutagenesis using PfSET7FL as template (primers in Table S1). Clones were verified to be error free and in frame by sequencing (GATC).

### Protein expression and purification

Recombinant proteins were expressed using a baculoviral expression system following manufacturer’s instructions. Briefly, 5 μg of plasmid, 500 ng linearised baculovirus DNA (BestBac 2.0, Expression Systems) was transfected with Cellfectin (Life Technologies) into Sf9 insect cells grown in media (Insect Xpress, Lonza) supplemented with 5% fetal bovine serum and 50 ug/ml gentamycin. Positive baculoviral clones were selected and viral stocks for protein production were made by several cycles of viral amplification. Recombinant proteins were purified using StrepTrap HP columns (GE Healthcare). Briefly, pellets from infected SF9 insect cells were resuspended in lysis buffer (10 mM TRIS, pH 7.5, 300 mM NaCl, 1% Triton X-100, 10% Glycerol and protease inhibitors), lysed by sonication using 6 bursts of 15 seconds (Vibra-Cell, Sonics and Materials) with a 1 minute pause on ice between bursts. The lysate was clarified by centrifugation (20 000 × *g*, Beckman Coulter) for 1h at 4 °C. The cleared lysate was filtered through 0.45μm filters (Durapore, Millipore), loaded onto the column, washed with wash buffer (10 mM TRIS, pH 8, 150 mM NaCl, 1 mM EDTA with protease inhibitors), then eluted (10 mM TRIS, pH 8, 150 mM NaCl, 1 mM EDTA and 2.5 mM desthiobiotin). Protein concentration was determined by Bradford Assay (Bio-Rad Protein Assay, Bio-Rad). Purified recombinant proteins were aliquoted, flash frozen in liquid nitrogen and stored at −80 °C until use. All proteins were judged to be >90% pure by SDS-PAGE.

### Nucleosome extraction

To isolate nuclei, HEK cell pellets were resuspended in lysis buffer (20 mM HEPES, pH 7.5, 250 mM sucrose, 4 mM MgCl_2_, 0.5% NP-40, 0.4 mM PMSF and 5 mM TCEP), homogenized with a dounce (Wheaton), then centrifuged at 2000 × *g* for 15 minutes at 4 °C. The nuclear pellet was suspended in wash buffer (20 mM HEPES, pH 7.5, 250 mM sucrose, 4 mM MgCl_2_, 0.4 mM PMSF and 5 mM TCEP), centrifuged and nuclei were resuspended in wash buffer. To 0.5 ml of suspended nuclei, 20 ml of 0.65 M NaCl-sodium phosphate buffer (0.65 M NaCl, 50 mM phosphate, pH 6.8, 0.4 mM PMSF and 5 mM TCEP) was added dropwise with constant stirring, then vortexed for 1 hour at 4 °C. The nuclear lysate was passed through a column loaded with pre-wet Hydroxylapatite Fast Flow (Calbiochem) then washed with 0.65 M NaCl-sodium phosphate buffer. Nucleosomes were isolated with elution buffer (2.5 M NaCl, 50 mM phosphate, pH 6.8, 0.4 mM PMSF and 5 mM TCEP). Fractions containing nucleosomes were pooled and buffer-exchanged (HiPrep Desalting, GE Healthcare) into 50 mM sodium phosphate, pH 6.8. Pooled fractions were concentrated to 1 mg/ml by centrifugation (Amicon Ultra, Millipore) and stored at −20 °C.

### *In vitro* methytransferase assay

Standard reactions were performed in a total volume of 25 μl in KMT buffer (50 mM TRIS, pH 8.8, 5 mM MgCl_2_, 4 mM dithiothreitol), with HEK nucleosomes (0.2 mg/ml final) as substrate, unless otherwise stated. For autoradiography, 2.5 μl of S-[^3^H-methyl]-adenosyl-L-methionine (^3^H-AdoMet) was added (3.7 μM final, 15 Ci/mmol, Perkin-Elmer). For kinetic characterization, ^3^H-AdoMet was diluted with unlabeled AdoMet (Sigma) to give a final concentration of 100 μM at 0.025 Ci/mmol. PfSET7 enzyme was used at 25 nM and reactions were incubated at room temperature for 1 hour unless otherwise stated. HKMT reactions at various pH values were performed using citrate-HEPES-CHES buffer (50 mM Tri-Sodium Citrate, 50 mM HEPES, 50 mM CHES, 5 mM MgCl_2_ and 4 mM DTT)^43^. 10 μl of each reaction was spotted in duplicate onto P81 Filters (Unifilter 96 well plate, Whatman), washed with 200 mM ammonium bicarbonate, dried, overlaid with scintillation fluid (Betaplate Scint, Perkin-Elmer) and read in a scintillation counter (MicroBeta2, Perkin-Elmer) and counts were converted to enzyme turnover. Analysis by fluorography was performed by resolving 20 μl of standard HKMT reactions (25 nM of PfSET7FL or G9a, 20 μg/ml of either BSA or nucleosomes, 2.5 μl of ^3^H-AdoMet at 15 Ci/mmol, in a total volume of 25 μl in KMT buffer) on 4–15% SDS-PAGE, the gel was rinsed in EN^3^HANCE (Perkin-Elmer) according to manufacturer's instructions, dried and exposed to film (BioMax MR Film, Carestream). Duplicate reactions were performed with unlabeled AdoMet, resolved on 4–15% SDS-PAGE and silver stained (Dodeca, Silver Stain Kit, Bio-Rad). Mutational analysis experiments tested enzymes at 25 nM and 100 nM.

### Histone extraction and LC-MS/MS

Deuterium labeled S-[^2^H_3_-methyl]-adenosyl-L-methionine (CD3-AdoMet) was synthesized using iodomethane-d3, AgOTf and HCOOH, according to the method of Stecher^44^. Nano-liquid chromatography tandem mass spectrometry (nanoLC-MS/MS) was utilised to identify sites of PfSET7 methylation on histone variants H3 and H4 based on the observation of mass shifts resulting from the incorporation of CD3 from CD3-AdoMet. Histones were acid extracted from the *in vitro* reaction buffer and processed for nanoLC-MS/MS analysis as previously described^45^. Briefly, the histone proteins were propionylated to label unmodified and monomethylated lysines with a propionyl group (+56 Da), which will result in a trypsin digest cleaving only at arginine residues. This produces the same histone peptide sequences regardless of any post-translational modifications present on lysine residues, and thus enables relative quantitation between different modifications on the same peptide based on signal intensity in the mass spectrometer. Subsequent propionylation of the N-termini of the corresponding histone peptides results in enhanced chromatographic resolution of isobaric (i.e. same mass) peptides (e.g. trimethylation [42.0469 Da] and acetylation [42.0106 Da] of lysine) based on their degree of hydrophobicity, thereby eliminating issues relating to signal interference and increasing confidence in PTM identification^46^. Approximately 1μg of histone peptides were chromatographically resolved using an Ultimate 3000 RS-LC-nano System (Dionex), with an Acclaim PepMap100, C18 stationary phase, 2 μm particle size, 100 Å pore size, 75 μm internal diameter x 15 cm length column (Thermo Fisher). The nanoLC gradient started at 1% B (5% H2O, 95% MeCN, and 0.1% formic acid) and 99% A (0.1% formic acid and 100% H_2_O) and increased to 30% B over 35 min followed by an increase to 95% B over 30 mins. Real-time tandem mass spectra were acquired on an LTQ Velos Pro linear ion trap (Thermo Scientific) with a 110 min acquisition time. Targeted zoom scans were performed for m/z values corresponding to the modified histone peptides to mitigate dynamic range issues and tandem mass spectra corresponding to methylated histone peptides were acquired using a priority queue.

### Transgenic parasite plasmid constructs

The CRISPR/Cas9 genome editing approach was employed to generate HA-tagged PfSET7 transgenic parasite line as described previously^47^. We used Protospacer Workbench to identify sgRNA sequences for PfSET7 and computationally predict off-target sites^48^. A 20-nt gRNA target sequence (5′-CACTGATGCTCCTCAAATTG-3′) directing Cas9 cleavage near the C-terminal end of PfSET7 was cloned into the sgRNA-expression cassette in the pL6 plasmid. Two homology regions were inserted in separate cloning steps using Gibson assembly to generate the pL6-Pfset7 plasmid. The primers used are listed in Table S1. A second plasmid, pUF1-Cas9, containing an engineered *S. pyogenes* endonuclease Cas9 cassette and regulatory elements of *P.falciparum* U6 snRNA was reported previously^47^.

### Parasite culture and transfections

Asexual blood-stage parasites were cultivated using standard methods, and synchronous cultures were obtained by sorbitol treatment and plasmion enrichment. Parasites were transfected using the pre-loaded red blood cells method^49^. Positive selection using 2.66 nM WR99210 and 1.5 μM DSM1 and negative selection using 40 μM 5-fluorocytosine were applied sequentially in culture until the integrant transfected line was established. Genomic integration was verified by PCR.

### PfSET7 antibody production

C57BL/6 and BALB/C mice were immunized 4 times at 14-day intervals with 20 ug doses of purified recombinant PfSET7-FL in Freund’s Complete Adjuvant for the initial injection and Freund’s Incomplete Adjuvant for booster injections to generate a polyclonal anti-PfSET7 antibody. Specificity of immune sera was determined by Western blot (Fig. S25).

### Subcellular fractionation and western blotting

Subcellular extract preparations were prepared as described previously^50^. Equal amounts of protein were loaded and separated on 4–12% SDS-PAGE gel (Invitrogen), and analyzed by western blot using antibodies anti-PfSET7 (mouse), anti-HA (mouse, Roche 12CA5 or rat, Roche 3F10), anti-Pf-aldolase (Abcam ab38905) or anti-Histone H3 (mouse, Abcam ab1791), all at 1:2000 dilution. Secondary antibodies were anti-rabbit IgG-HRP (GE NA934V, Lot 9568295) and anti-mouse IgG-HRP (GE NA931V, Lot 9648752), both at 1:4000. Blots were developed using ECL (Thermo Scientific).

### Immunofluorescence microscopy

Immunofluorescence microscopy on extracellular and intracellular parasites was performed as described previously^51^. Antibody dilutions are as follows: anti-PfHP1 (rabbit, Genscript) 1:4000, anti-PfSET7 (mouse) 1:2000, anti-HA (mouse or rat, Roche) 1:2000, anti-Bip (rabbit, MR4) 1:1000, anti-ERD2 (rat, MR4) 1:1000, and secondary goat anti-rat/rabbit/mouse Alexa-Fluor 488 or 568 conjugates (Invitrogen), all at 1:2500. Mitochondria were stained with 100 nM MitoTracker-DeepRedFM for 20 minutes at 37 °C in the dark before fixation and permeabilization. Images were captured using a Nikon Eclipse 80i microscope with a CoolSnapHQ2 camera (Photometrics). NIS Elements version 3.0 software was used for image acquisition, and Fiji software was used for analysis.

*P. falciparum* (NF54) sporozoites were isolated by aseptic dissection of the salivary glands of infected *Anopheles stephensi* obtained from the Department of Medical Microbiology, University Medical Centre, St Radboud, Nijmegen, the Netherlands. 5 × 10^4^ sporozoites placed on poly-L-lysine coated coverslips were either fixed or activated for 1 h at 37 °C and then fixed in 4% PFA for 10 min at room temperature. Samples were blocked in normal goat serum diluted 1:500 for 2 h at 37 °C, washed twice in PBS and incubated with anti-PfSET7 antibody at 1:1000 and anti-PfHP1 at 1:2000 for 1 h, washed twice in PBS, then incubated with secondary goat anti-mouse IgG coupled to Alexa-Fluor 488 or 594 diluted 1:500 with DAPI 1 μg/ml in the dark for 1 h.

Fifty micron thick serial sections of frozen liver from humanized TK-NOG mice infected with *P. falciparum* were prepared as previously described^52^. Sections were incubated overnight with anti-PfSET7 antibody diluted 1:1000 and anti-PfHP1 antibody diluted 1:2000, washed twice in PBS, then incubated with secondary antibodies and DAPI as above, at 37 °C. All antibodies were diluted in PBS containing 1% BSA and 0.2% Triton X-100. All sections were washed twice in PBS before being mounted in anti-fading medium and stored at 4  °C before analysis. Immunostained sporozoites and liver sections were examined under a confocal microscope (Olympus FV-1000, Plateforme d’Imagerie Cellulaire PICPS, La Pitié-Salpêtrière, Paris) and images were analysed using ImageJ software.

### Ethics Statement

All animal care and experiments involving mice were conducted at the Institut Pasteur, approved by the ‘Direction Départementale des Services Vétérinaires’ de Paris, France (Permit Number N° 75–066 issued on September 14, 2009) and performed in compliance with institutional guidelines and European regulations (http://ec.europa.eu/environment/chemical?s/lab_animals/home_en.htm). A statement of compliance with the French Government’s ethical and animal experiment regulations was issued by the Ministère de l’Enseignement Supérieur et de la Recherche under the number 00218.01.

## Additional Information

**How to cite this article**: Chen, P. B. *et al.* *Plasmodium falciparum* PfSET7: enzymatic characterization and cellular localization of a novel protein methyltransferase in sporozoite, liver and erythrocytic stage parasites. *Sci. Rep.* **6**, 21802; doi: 10.1038/srep21802 (2016).

## Supplementary Material

Supplementary Information
