# Supplementary material for: Plasmodium falciparum PfSET7: enzymatic characterization and cellular localization of a novel protein methyltransferase in sporozoite, liver and erythrocytic stage parasites
Source: Sci Rep. 2016 Feb 23;6:21802. doi: 10.1038/srep21802 (PMC4763181; doi:10.1038/srep21802)
Supplement: Supplementary Information [file srep21802-s1.pdf]

Title

Plasmodium falciparum PfSET7: enzymatic characterization and cellular localization of a novel protein methyltransferase in sporozoite, liver and erythrocytic stage parasites

Authors

Patty B. Chen[1,2,3,§], Shuai Ding[1,2,3,§], Gigliola Zanghì[4], Valérie Soulard[4], Peter A. DiMaggio Jr[6], Matthew J. Fuchter[7], Salah Mecheri[1,2,3], Dominique Mazier[4,5], Artur Scherf[1,2,3], Nicholas A. Malmquist[1,2,3,*]

[1] Unité Biologie des Interactions Hôte-Parasite, Département de Parasites et Insectes Vecteurs, Institut Pasteur, Paris 75015, France. [2] CNRS, ERL 9195, Paris 75015, France. [3] INSERM, UMR 1201, Paris 75015, France. [4] Sorbonne Universités, UPMC Univ Paris 06, INSERM U1135, CNRS ERL 8255, Centre d'Immunologie et des Maladies Infectieuses (CIMI-Paris), 91 Bd de l'hôpital, 75013, Paris, France. [5] AP HP, Centre Hospitalo-Universitaire Pitié-Salpêtrière, 75013 Paris, France. [6] Department of Chemical Engineering, Imperial College London, South Kensington Campus, London SW7 2AZ, United Kingdom. [7] Department of Chemistry, Imperial College London, South Kensington Campus, London SW7 2AZ, United Kingdom.

[*]Correspondence and requests for materials should be addressed to N.A.M (e-mail: nicholas.malmquist@pasteur.fr)

[§]These authors contributed equally to this work.

Supplementary Data

Product	Primer	Sequence
**PfSET7ΔC**	**Primer 1F**<br>**Primer 3R**	**5'-CATCGGGCGCGGATCCATGGAAACTATTTTTAGGAAACTC-3'**<br>**5'-ATAGAGGTCATCGTCGCCCAGGTA-3'**
**PfSET7ΔCΔN**	**Primer 2F**<br>**Primer 3R**	**5'-CATCGGGCGCGGATCCATGAACGACGTGGAAATCTTCAATGTCA-3'**<br>**5'-ATAGAGGTCATCGTCGCCCAGGTA-3'**
**PfSET7ΔN**	**Primer 2F**<br>**Primer 5R**	**5'-CATCGGGCGCGGATCCATGAACGACGTGGAAATCTTCAATGTCA-3'**<br>**5'-AGATCTGCAGCGGCCGGATCAGCGGGTTTAAACTCACTTCTC-3'**
**Strep-Tag**	**Primer 4F**<br>**Primer 5R**	**5'-GACGATGACCTCTATGACGATGACGATAAGGCCGGTTG-3'**<br>**5'-AGATCTGCAGCGGCCGGATCAGCGGGTTTAAACTCACTTCTC-3'**
**PfSET7_H517A**	**Primer 6F**<br>**Primer 7R**	**5'-CGCATCTCTATGCTGGCAGCCTCATGCATTAGTACAGCCTG-3'**<br>**5'-CAGGCTGTACTAATGCATGAGGCTGCCAGCATAGAGATGCG-3'**
**pL6-Pfset7 plasmid Homology Region 1**	**Primer 8F**<br>**Primer 9R**	**5'-TTCCGCGGGGAGGACTAGTCCAATTATTTCCAAGTGCAATGT-3'**<br>**5'-TATCCACCGCCTGGCGCGCCCTCTTCGATCTGAGGAGCAT-3'**
**pL6-Pfset7 plasmid Homology Region 2**	**Primer 10F**<br>**Primer 11R**	**5'-TGTAGGAGGGGACGGCGCCATATATTTAGCAATAACTTC-3'**<br>**5'-AATTTTTTTTACAAAATGCTTAAGTACCTTAGAACAAATAGG-3'**
**Integration confirmation**	**Primer 12F**<br>**Primer 13R**<br>**Primer 14F**<br>**Primer 15R**	**5'-GTATGTATGTGTAGTAGGTGTACTCATCC-3'**<br>**5'-GCATAGTCTGGTACGTCATAGGGATACG-3'**<br>**5'-CATGGGGACAACAATGGGGAGAAAGAGG-3'**<br>**5'-GCTTAGTTGACGAGGATGGAGGTTATCG-3'**
**PfSET7 qPCR**	**Primer qF**<br>**Primer qR**	**5'-GATTTGTCTGATGCCTTAGC-3'**<br>**5'-TTTCCCATTGAGATGTATCC-3'**

Table S1 I **Primers for generating PfSET7 mutants, pL6-Pfset7 constructs and quantitative PCR.**

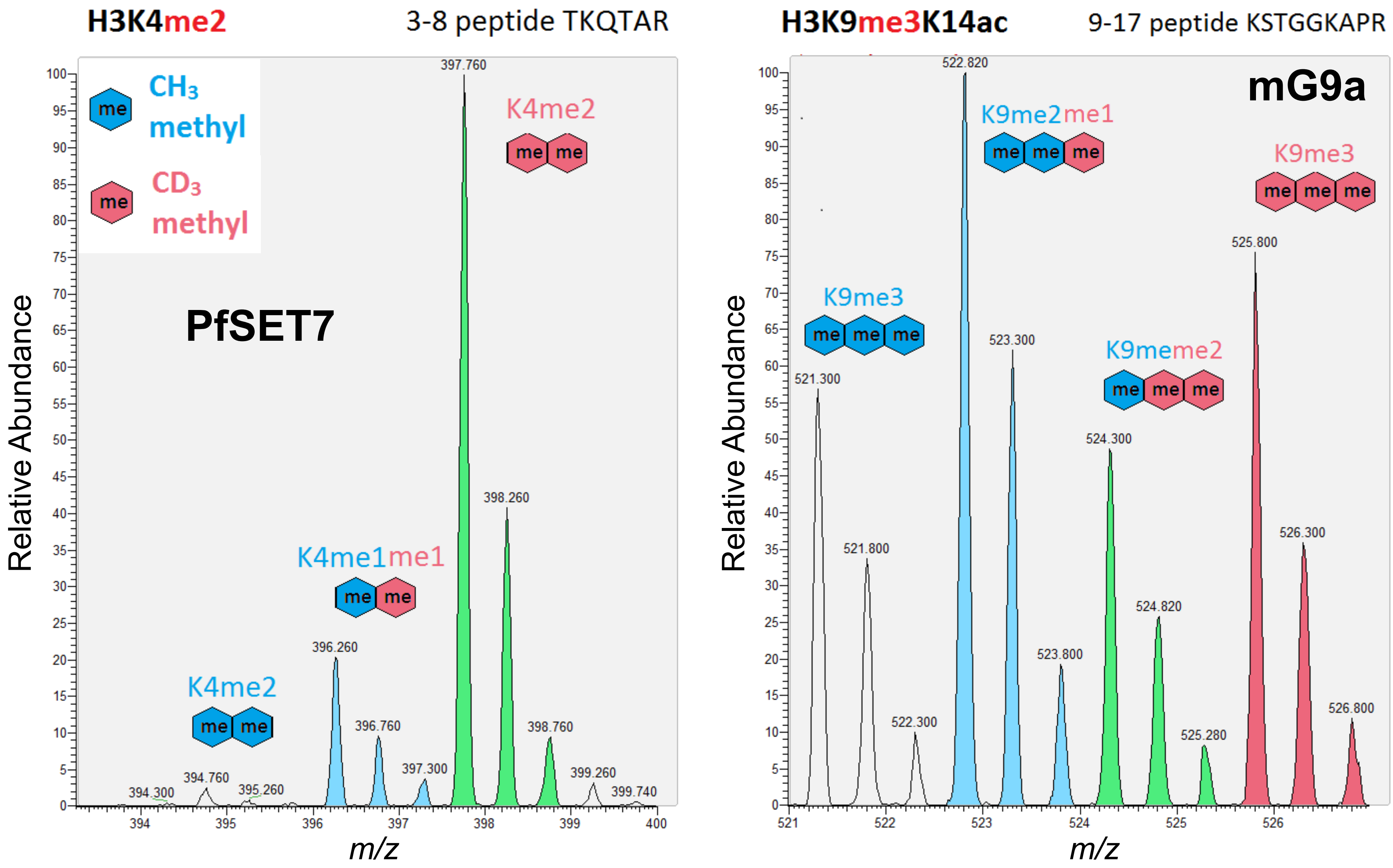

Figure S1 | Left Panel: PfSET7 methylation of H3K4me2, where the most abundant product is H3K4 with two new CD3 methyl groups (green series of isotopic peaks). Right Panel: heavy methyl labeling observed for mouse G9a-mediated methylation of H3K9me3K14ac.

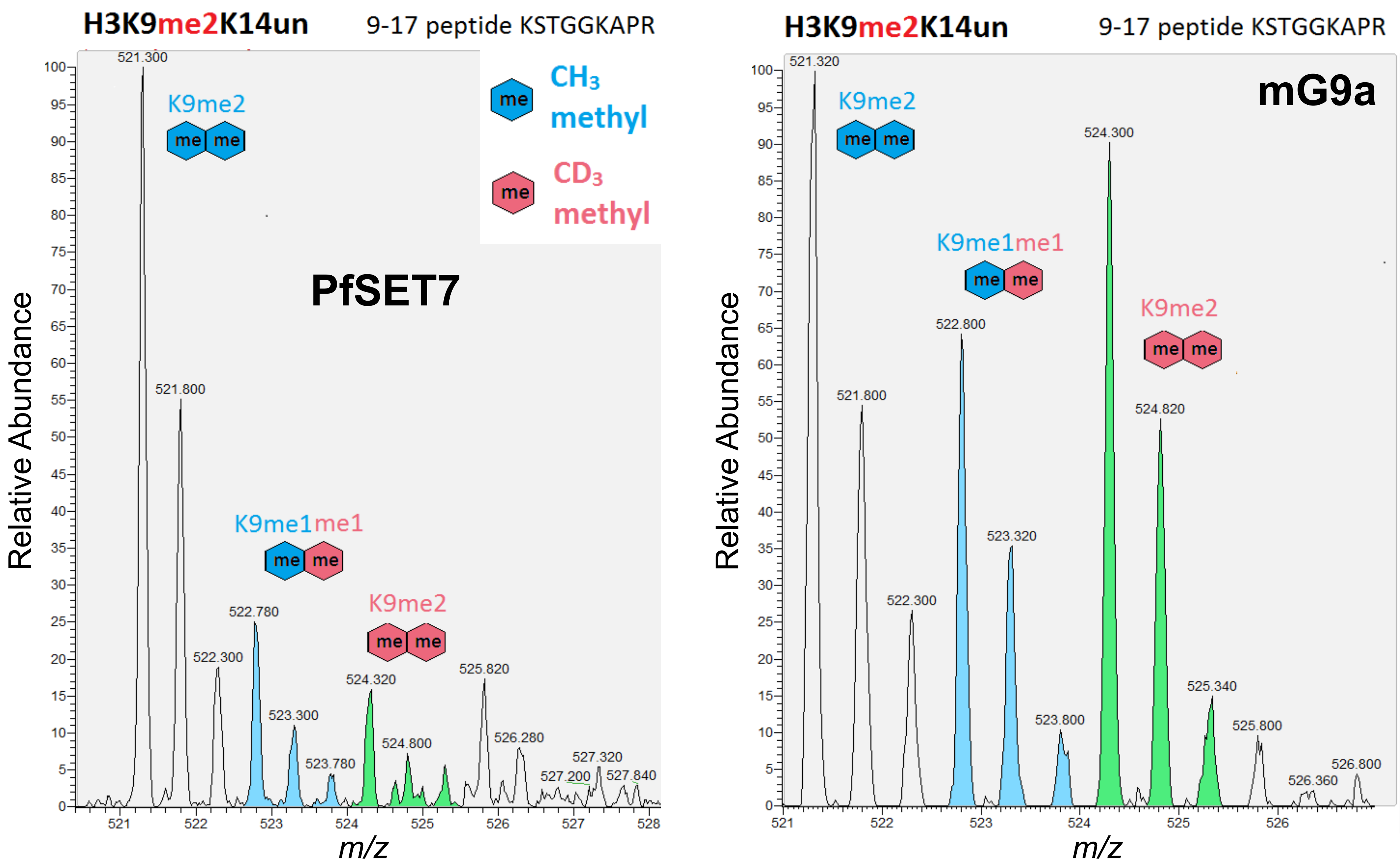

Figure S2 | Left Panel: PfSET7 methylation of H3K9me2K14un, which is relatively low in abundance compared to substrates containing H3K14ac (compare to Fig. 5). Right Panel: heavy methyl labeling observed for mouse G9a-mediated methylation of H3K9me2K14un.

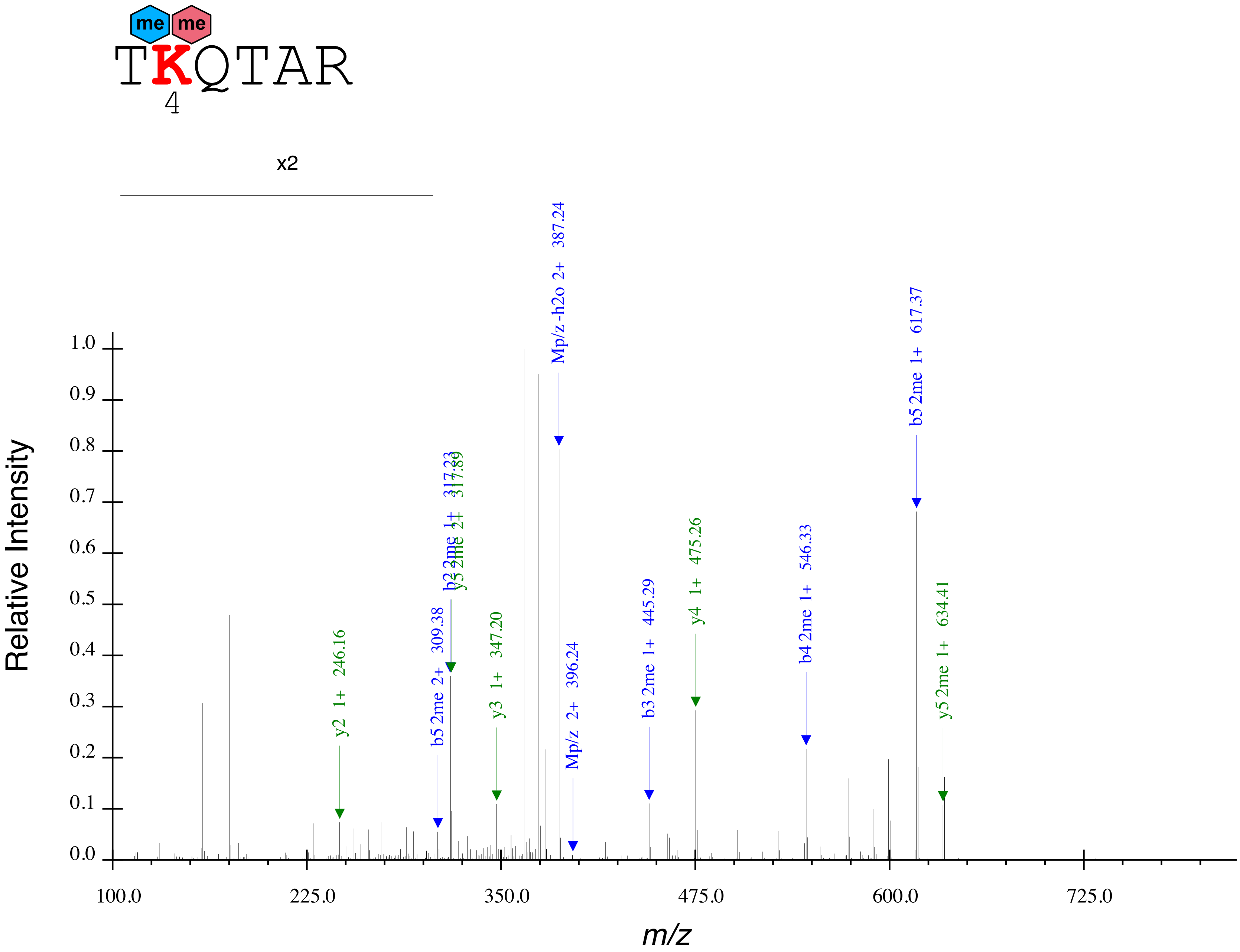

Figure S3 | Annotated CID tandem mass spectrum for H3K4me2(CH_3,CD_3), z = 2, Mp/z = 396.24. N-terminus has been propionylated (+56.0627 Da).

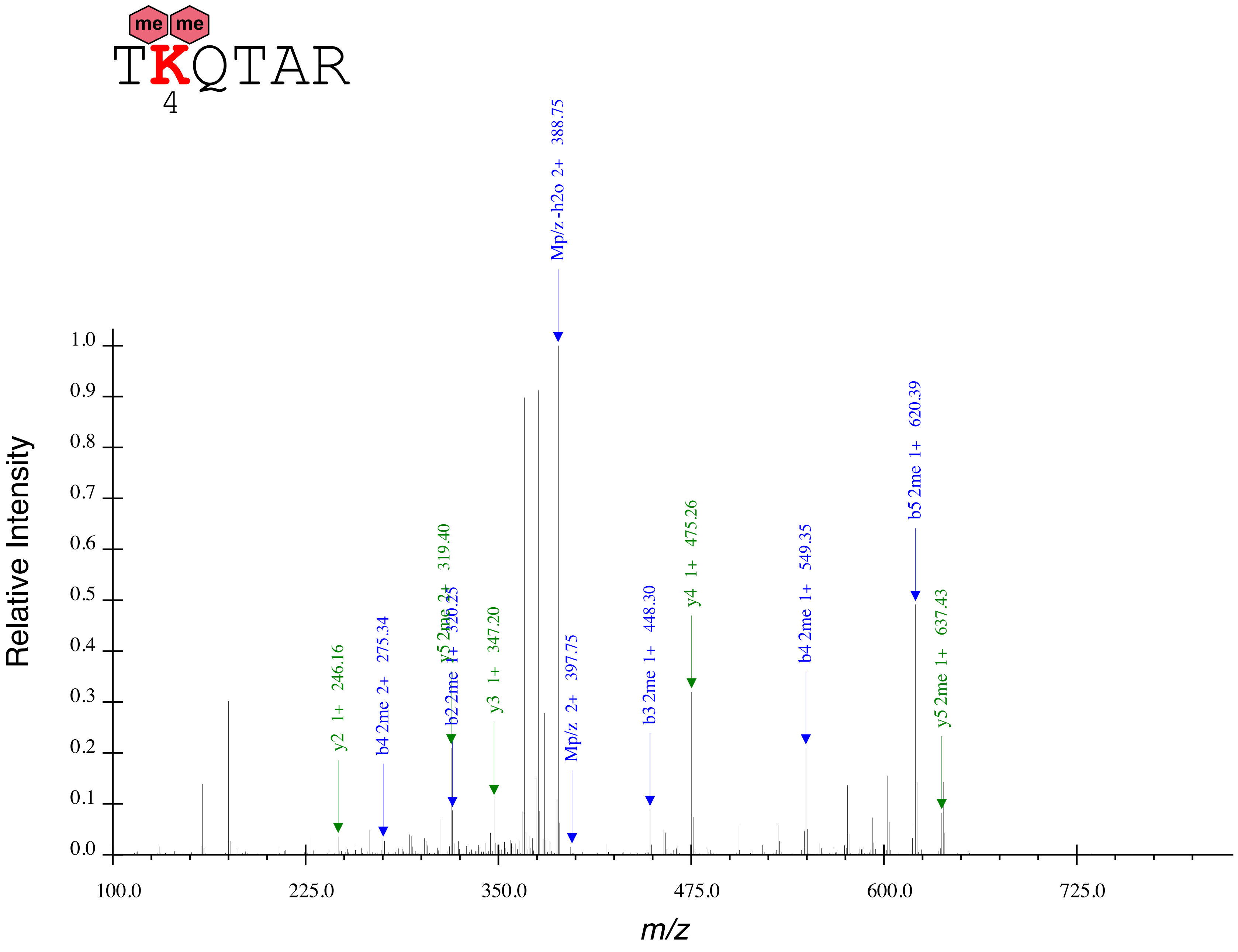

Figure S4 | Annotated CID tandem mass spectrum for H3K4me2(2CD_3), z = 2, Mp/z = 397.75. N-terminus has been propionylated (+56.0627 Da).

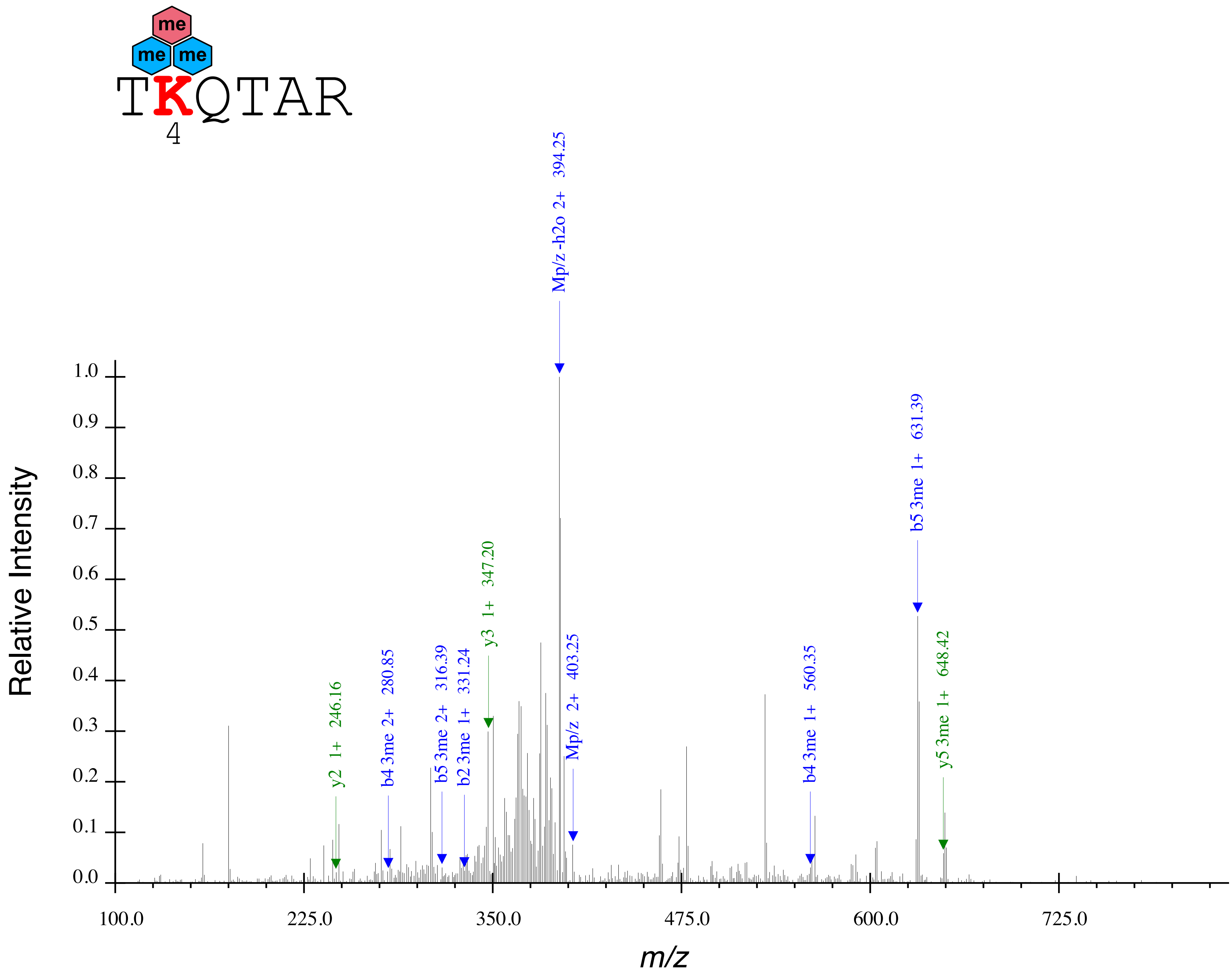

Figure S5 | Annotated CID tandem mass spectrum for H3K4me3(2CH_3,CD_3), z = 2, Mp/z = 403.25. N-terminus has been propionylated (+56.0627 Da).

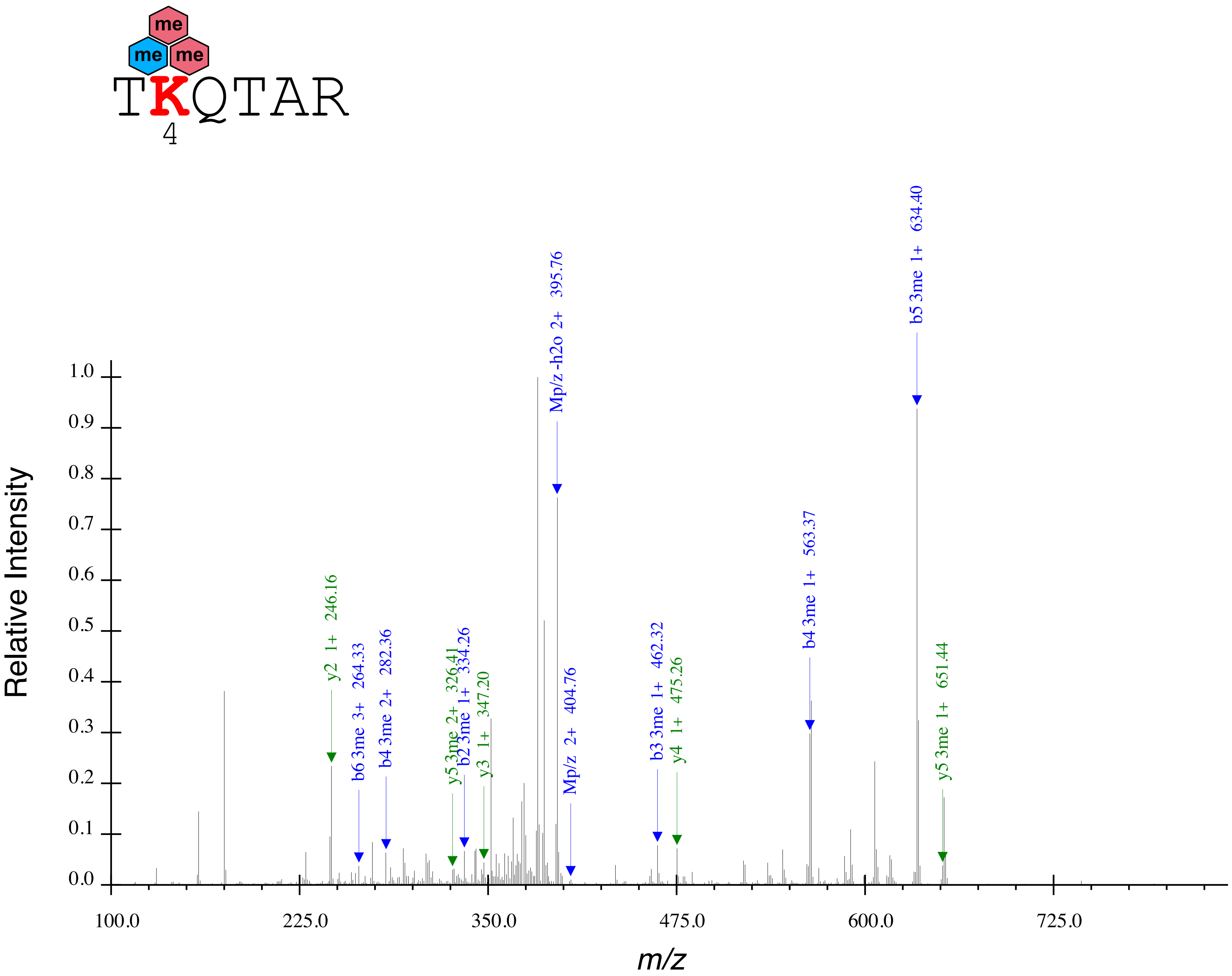

Figure S6 | Annotated CID tandem mass spectrum for H3K4me3(CH_3,2CD_3), z = 2, Mp/z = 404.76. N-terminus has been propionylated (+56.0627 Da).

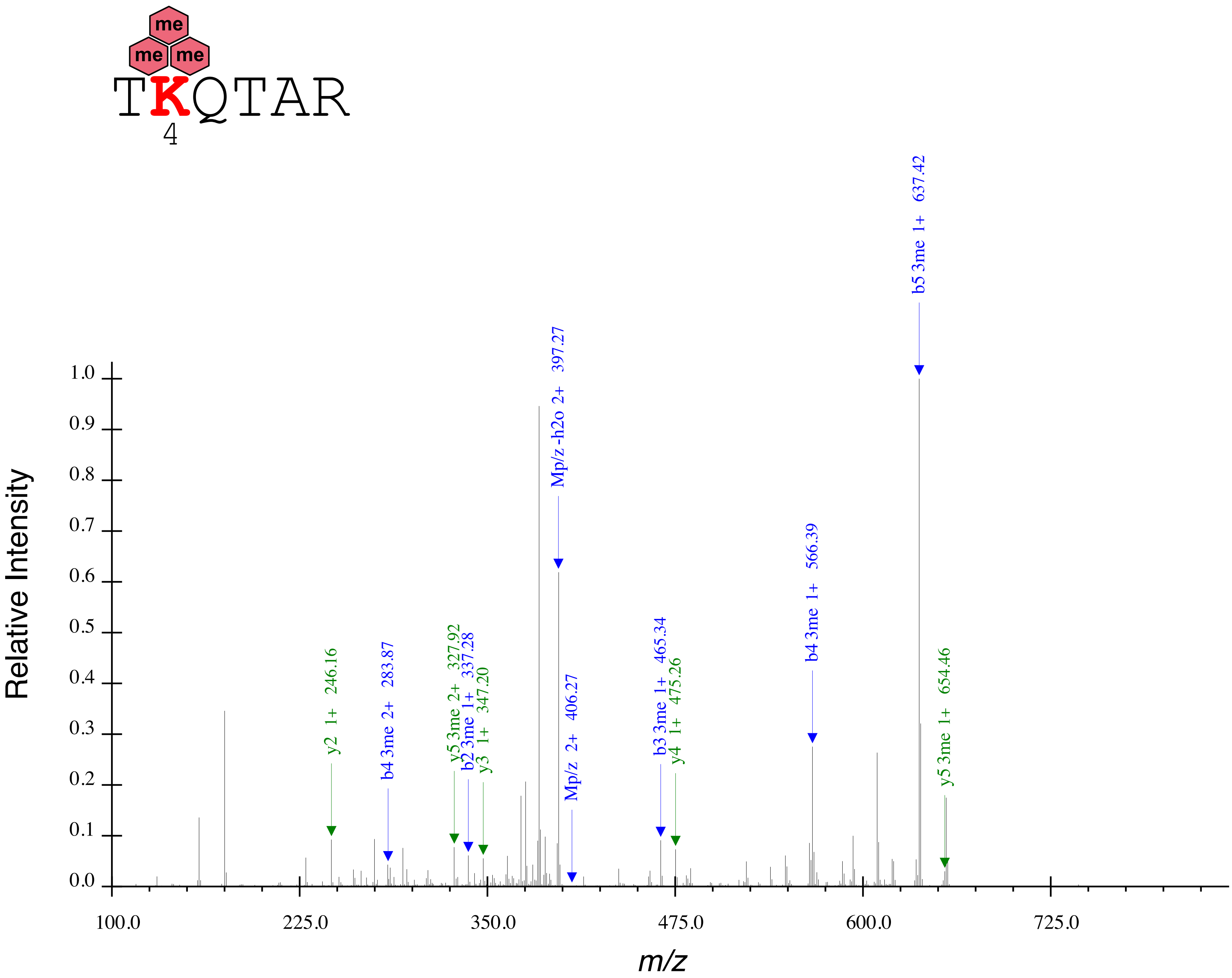

Figure S7 | Annotated CID tandem mass spectrum for H3K4me3(3CD_3), z = 2, Mp/z = 406.27. N-terminus has been propionylated (+56.0627 Da).

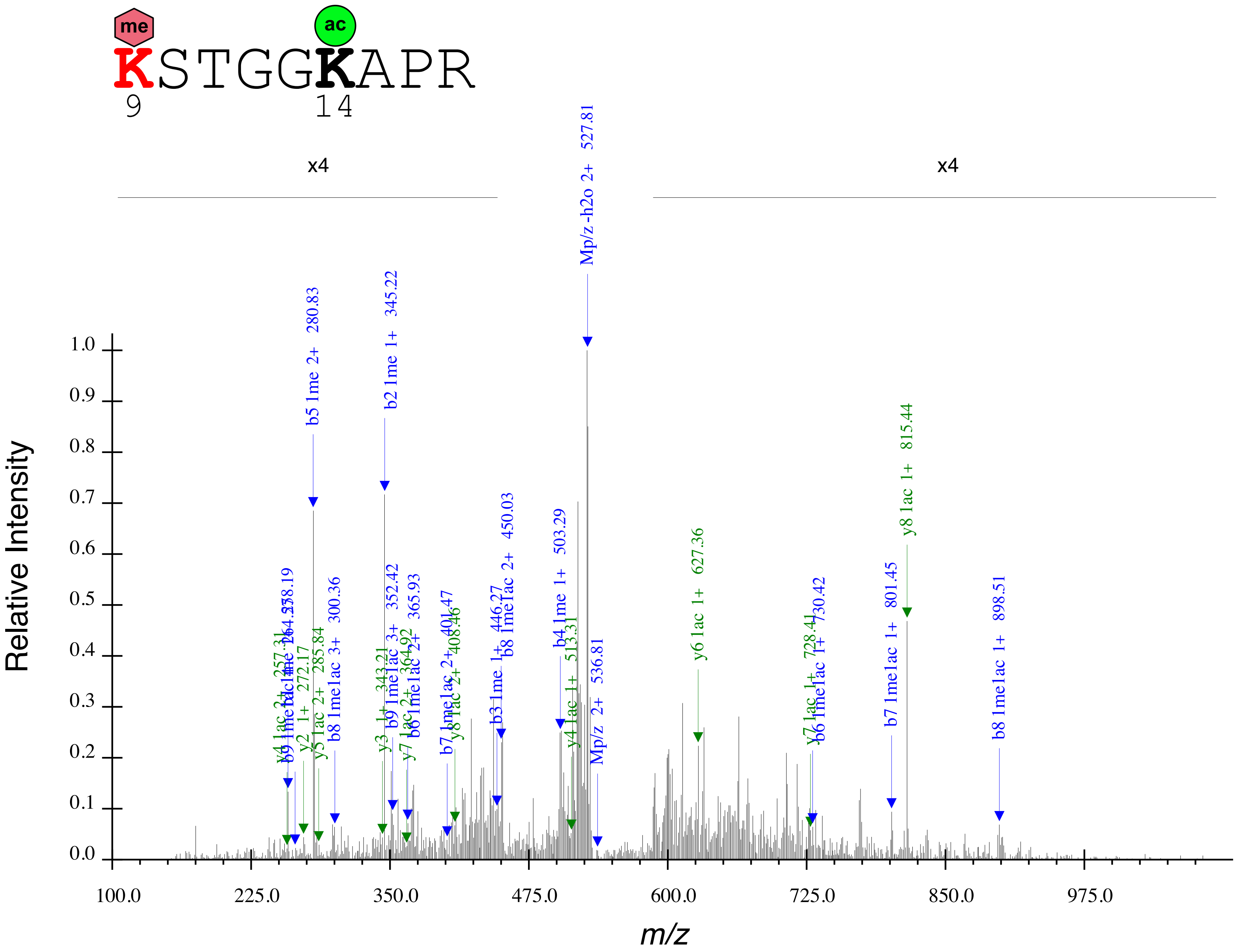

Figure S8 | Annotated CID tandem mass spectrum for H3K9me1(CD_3)K14ac, z = 2, Mp/z = 536.81. N-terminus and K9 have been propionylated (+56.0627 Da).

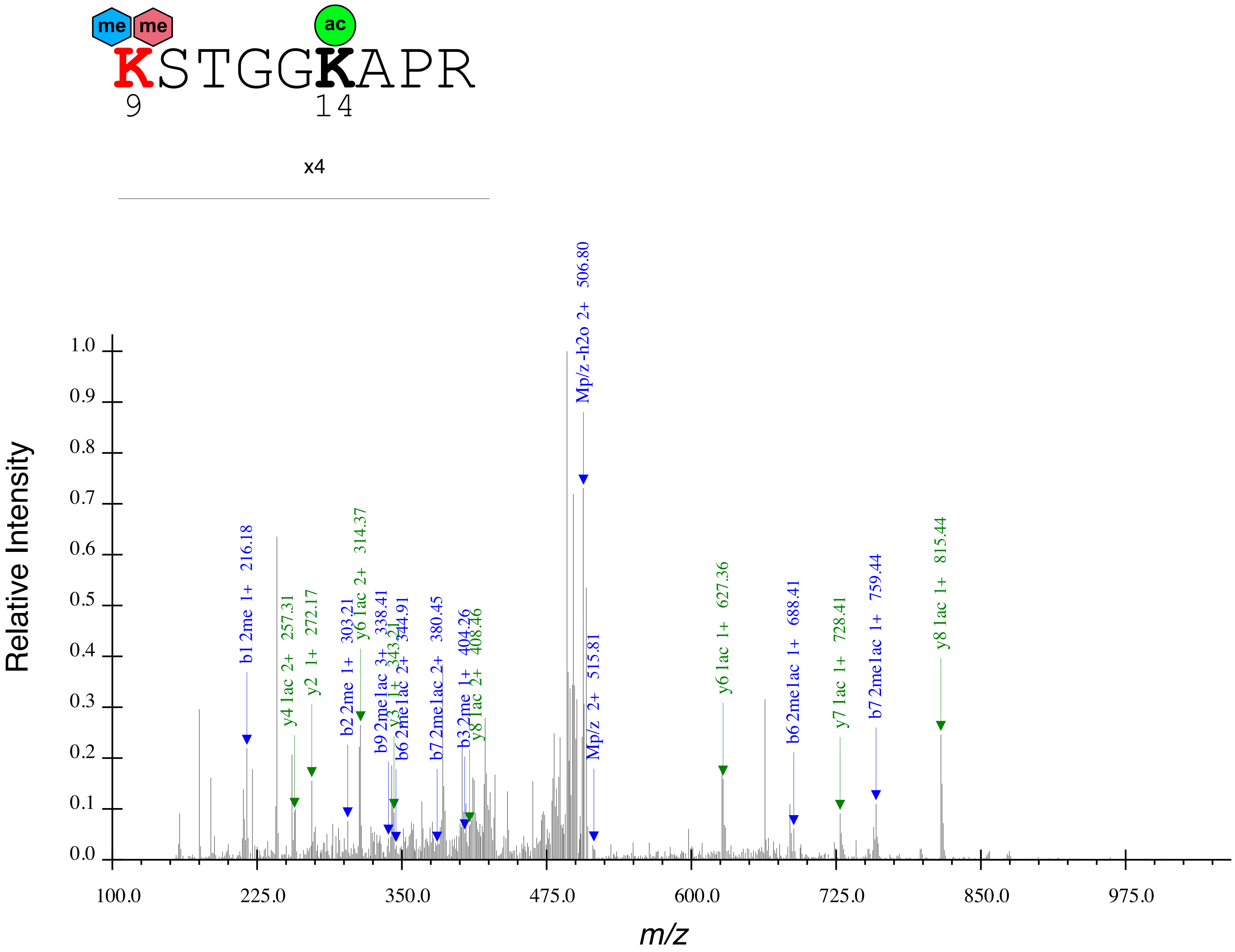

Figure S9 | Annotated CID tandem mass spectrum for H3K9me2(CH_3,CD_3)K14ac, z = 2, Mp/z = 515.81. N-terminus has been propionylated (+56.0627 Da).

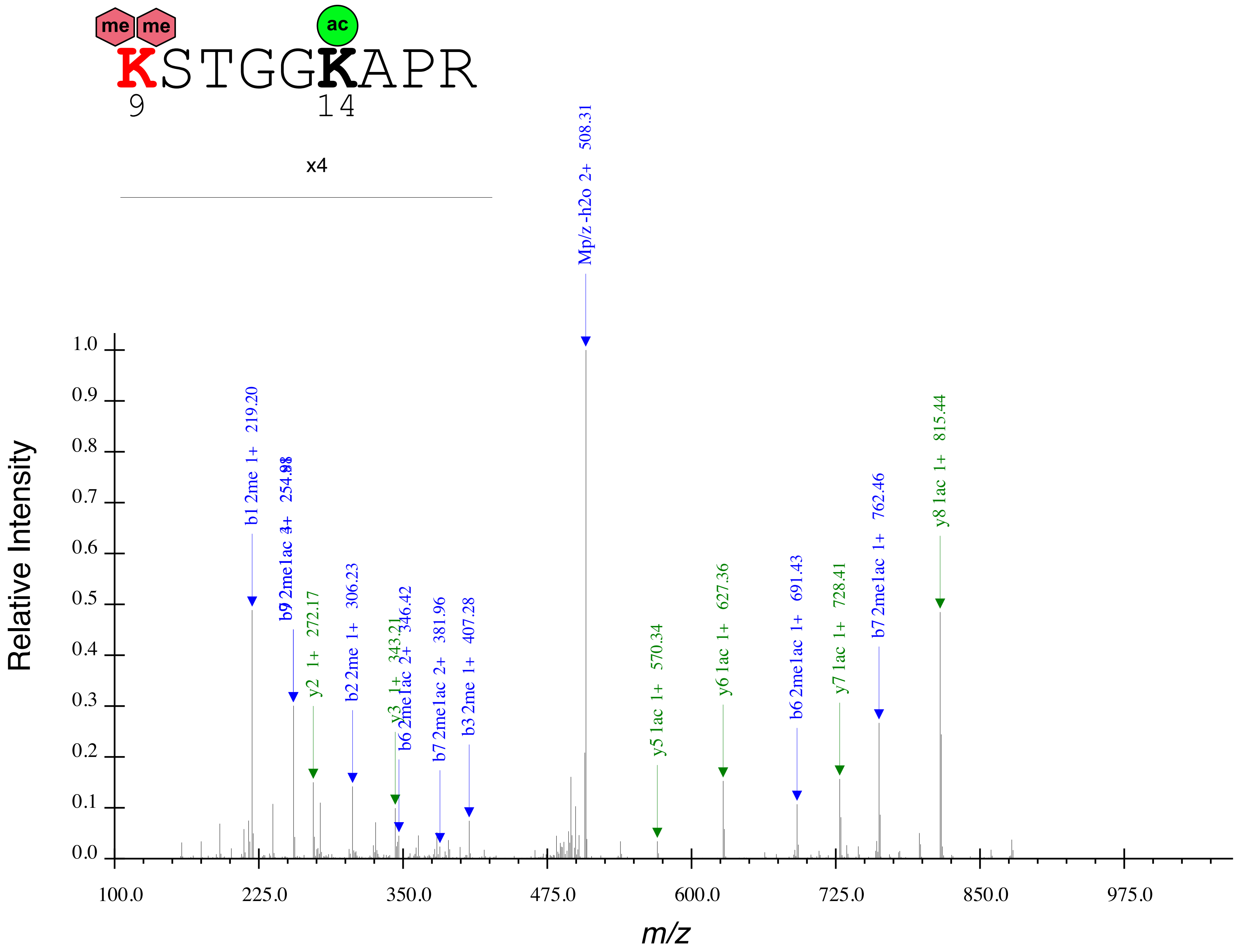

Figure S10 | Annotated CID tandem mass spectrum for H3K9me2(2CD_3)K14ac, z = 2, Mp/z = 517.31. N-terminus has been propionylated (+56.0627 Da).

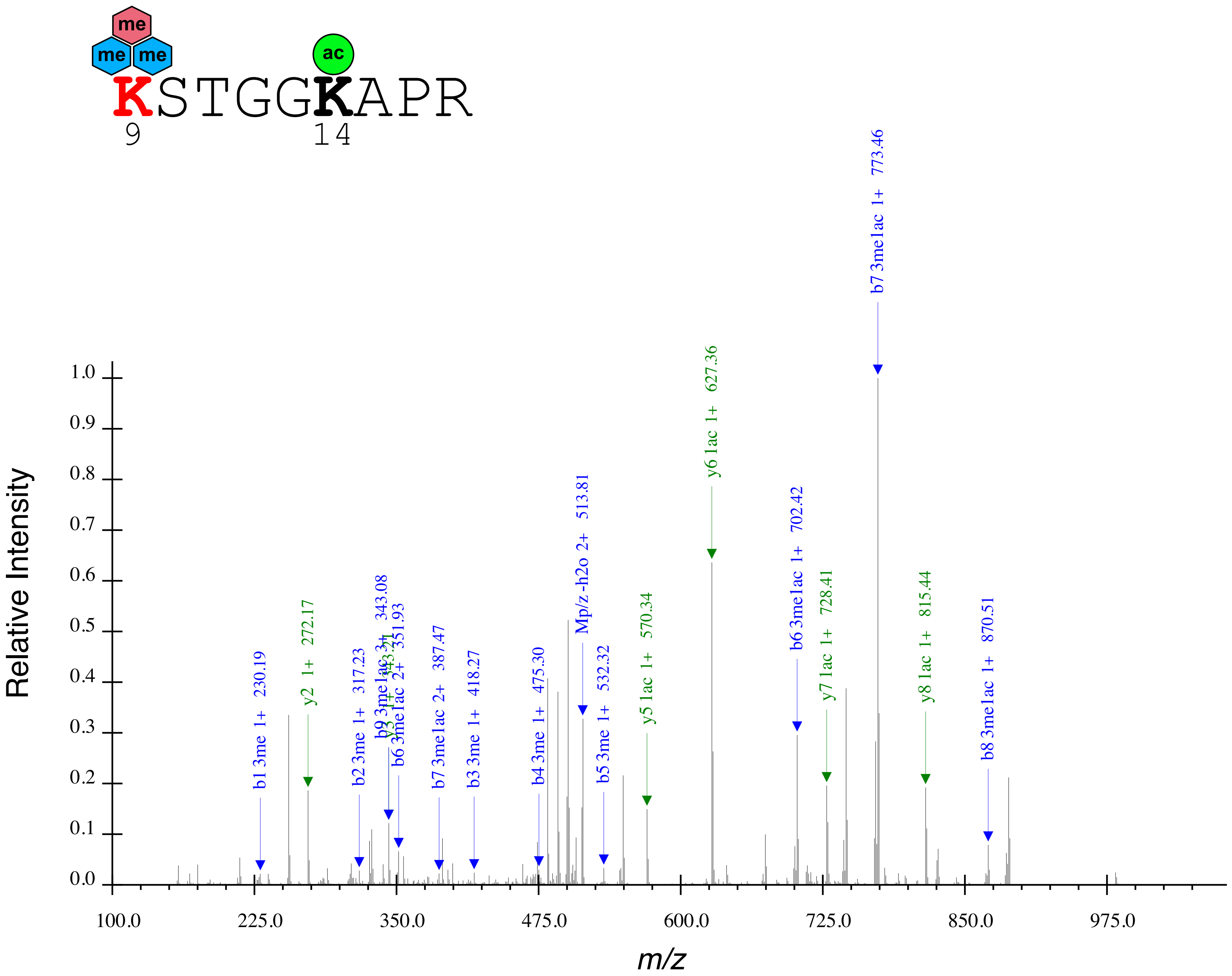

Figure S11 | Annotated CID tandem mass spectrum for H3K9me3(2CH_3,CD_3)K14ac, z = 2, Mp/z = 522.82. N-terminus has been propionylated (+56.0627 Da).

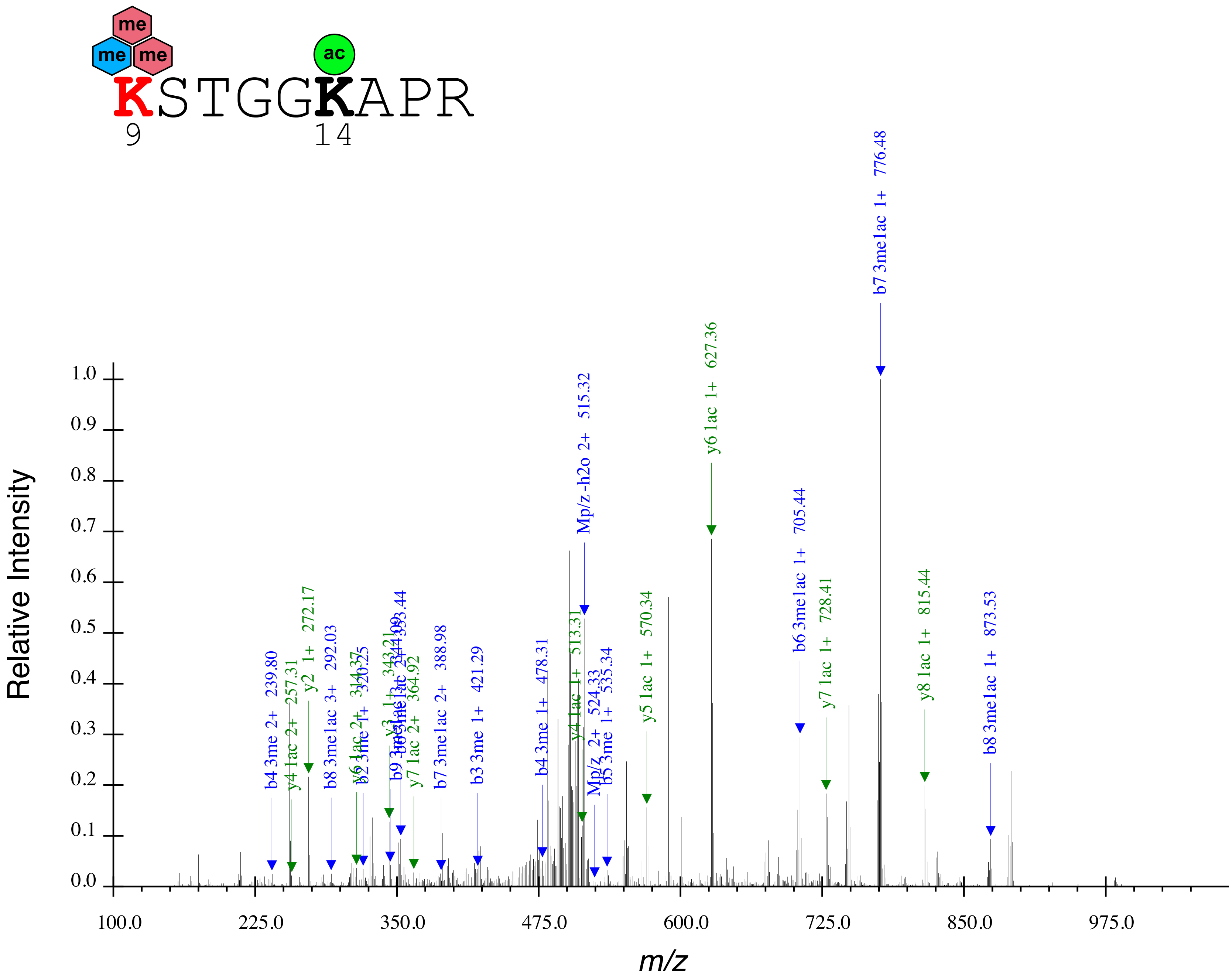

Figure S12 | Annotated CID tandem mass spectrum for H3K9me3(CH_3,2CD_3)K14ac, z = 2, Mp/z = 524.33. N-terminus has been propionylated (+56.0627 Da).

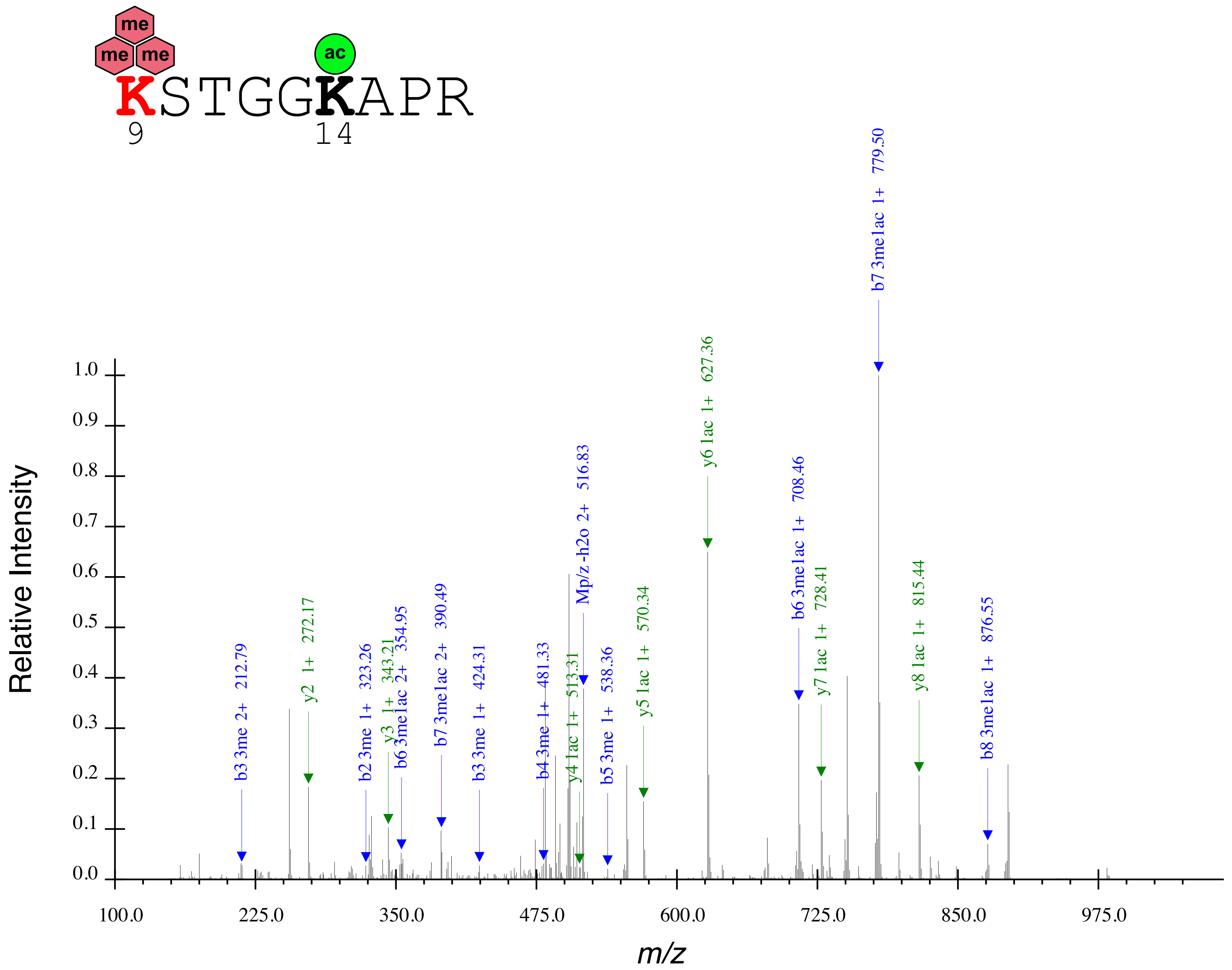

Figure S13 | Annotated CID tandem mass spectrum for H3K9me3(3CD_3)K14ac, z = 2, Mp/z = 525.84. N-terminus has been propionylated (+56.0627 Da).

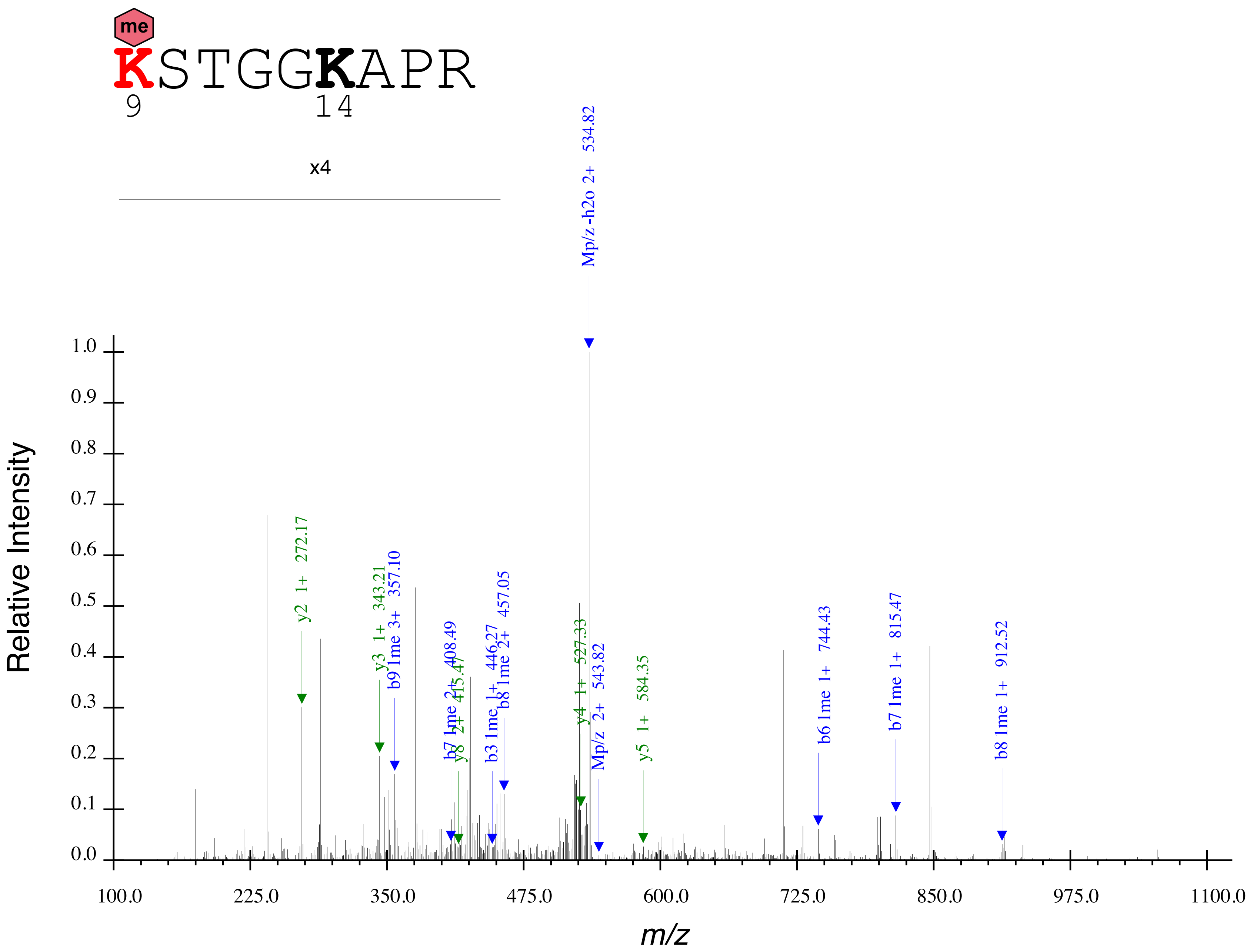

Figure S14 | Annotated CID tandem mass spectrum for H3K9me1(CD_3)K14un, z = 2, Mp/z = 543.82. N-terminus, K9 and K14 have been propionylated (+56.0627 Da).

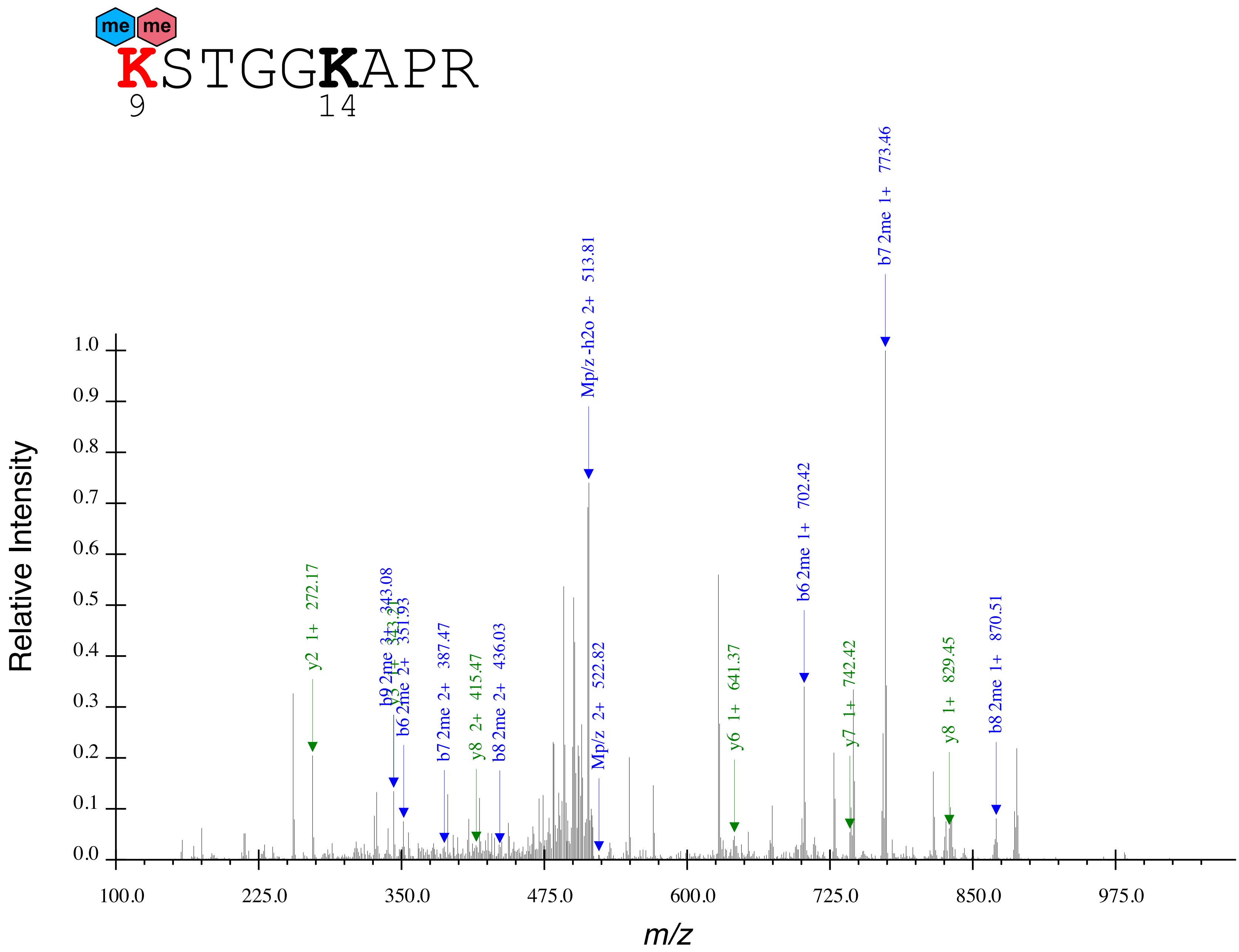

Figure S15 | Annotated CID tandem mass spectrum for H3K9me2(CH_3,CD_3)K14un, z = 2, Mp/z = 522.82. N-terminus and K14 have been propionylated (+56.0627 Da).

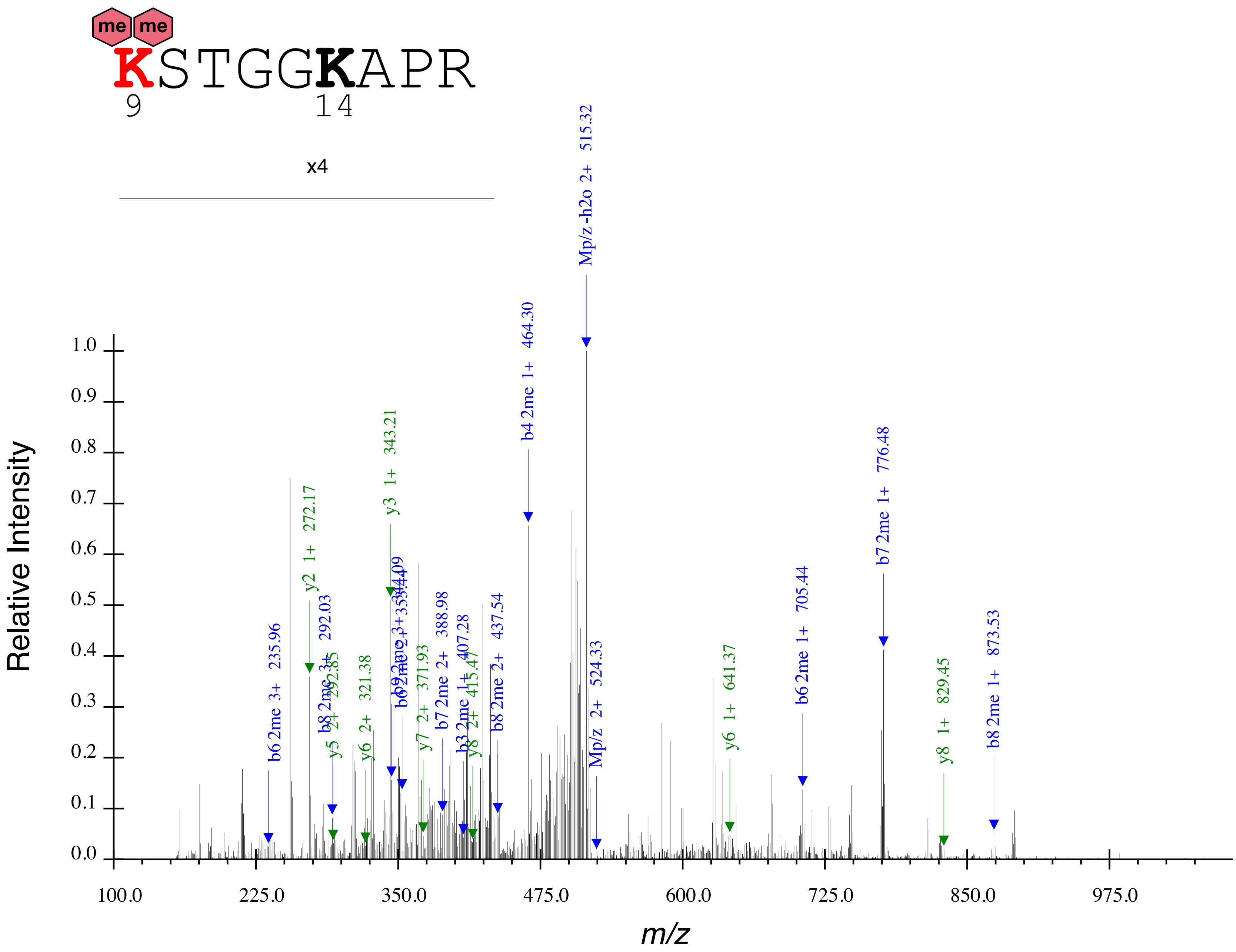

Figure S16 | Annotated CID tandem mass spectrum for H3K9me2(2CD_3)K14un, z = 2, Mp/z = 524.33. N-terminus and K14 have been propionylated (+56.0627 Da).

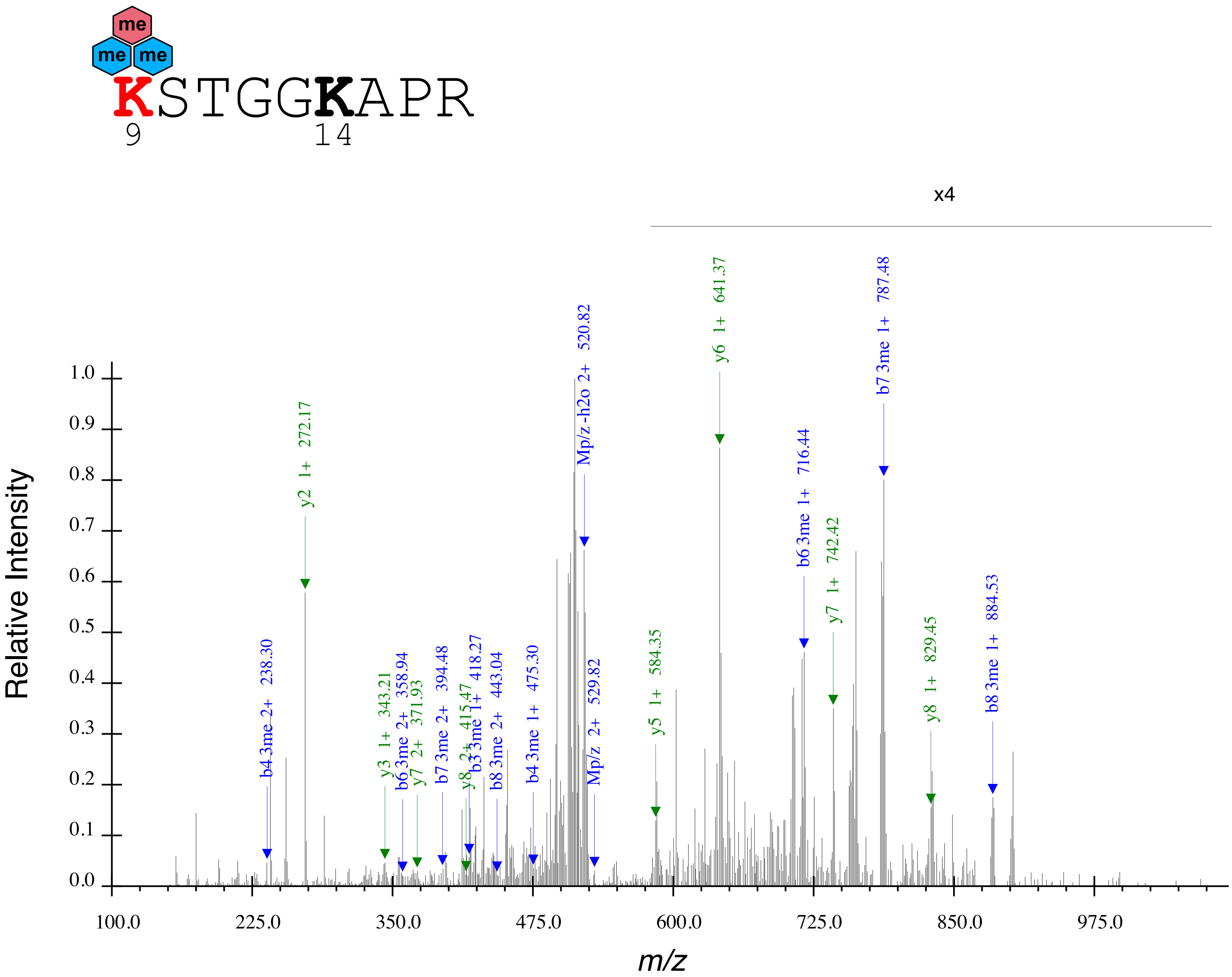

Figure S17 | Annotated CID tandem mass spectrum for H3K9me3(2CH_3,CD_3)K14un, z = 2, Mp/z = 529.82. N-terminus and K14 have been propionylated (+56.0627 Da).

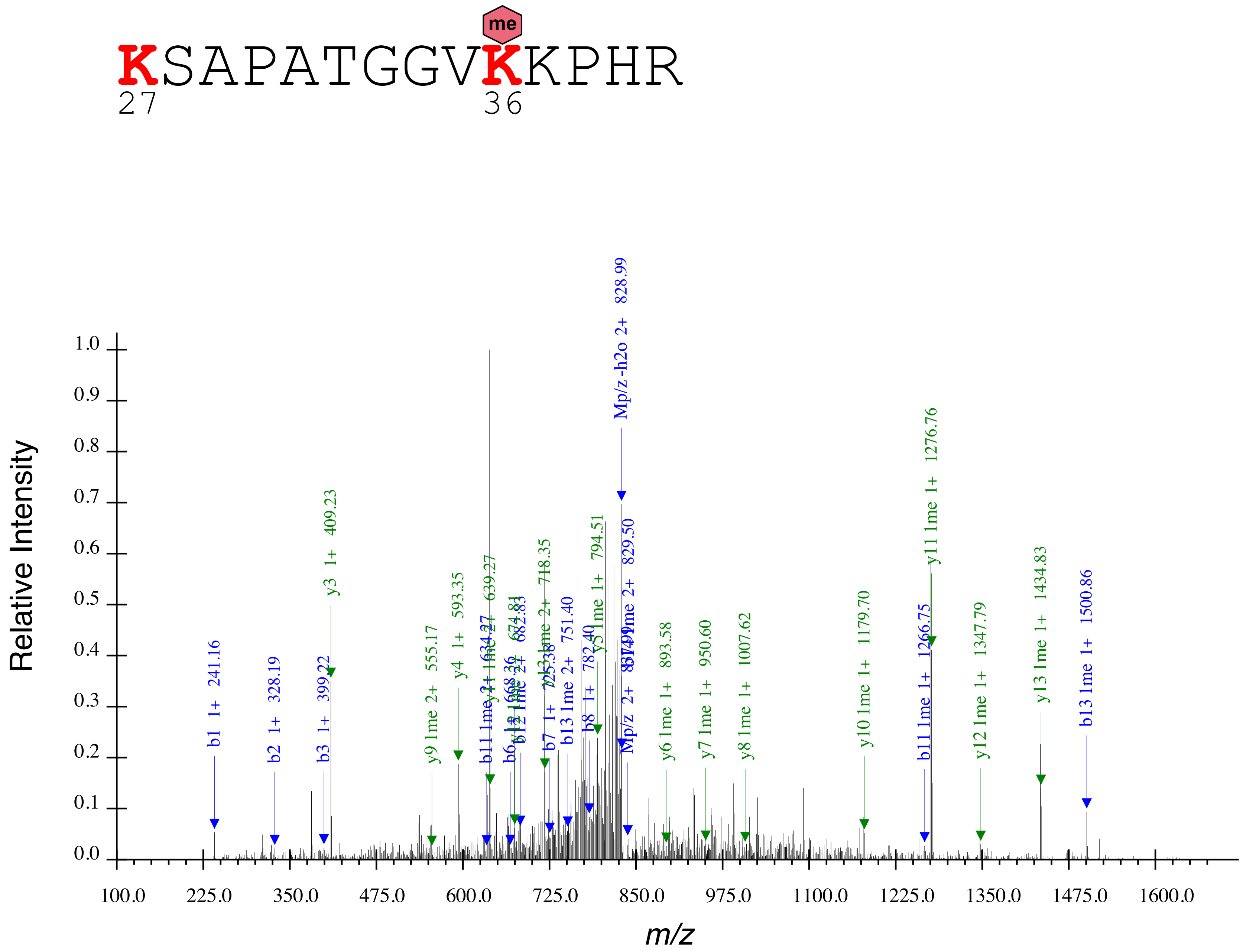

Figure S18 | Annotated CID tandem mass spectrum for H3K27unK36me1(CD_3), z = 2, Mp/z = 837.99. N-terminus, K27, K36 and K37 have been propionylated (+56.0627 Da).

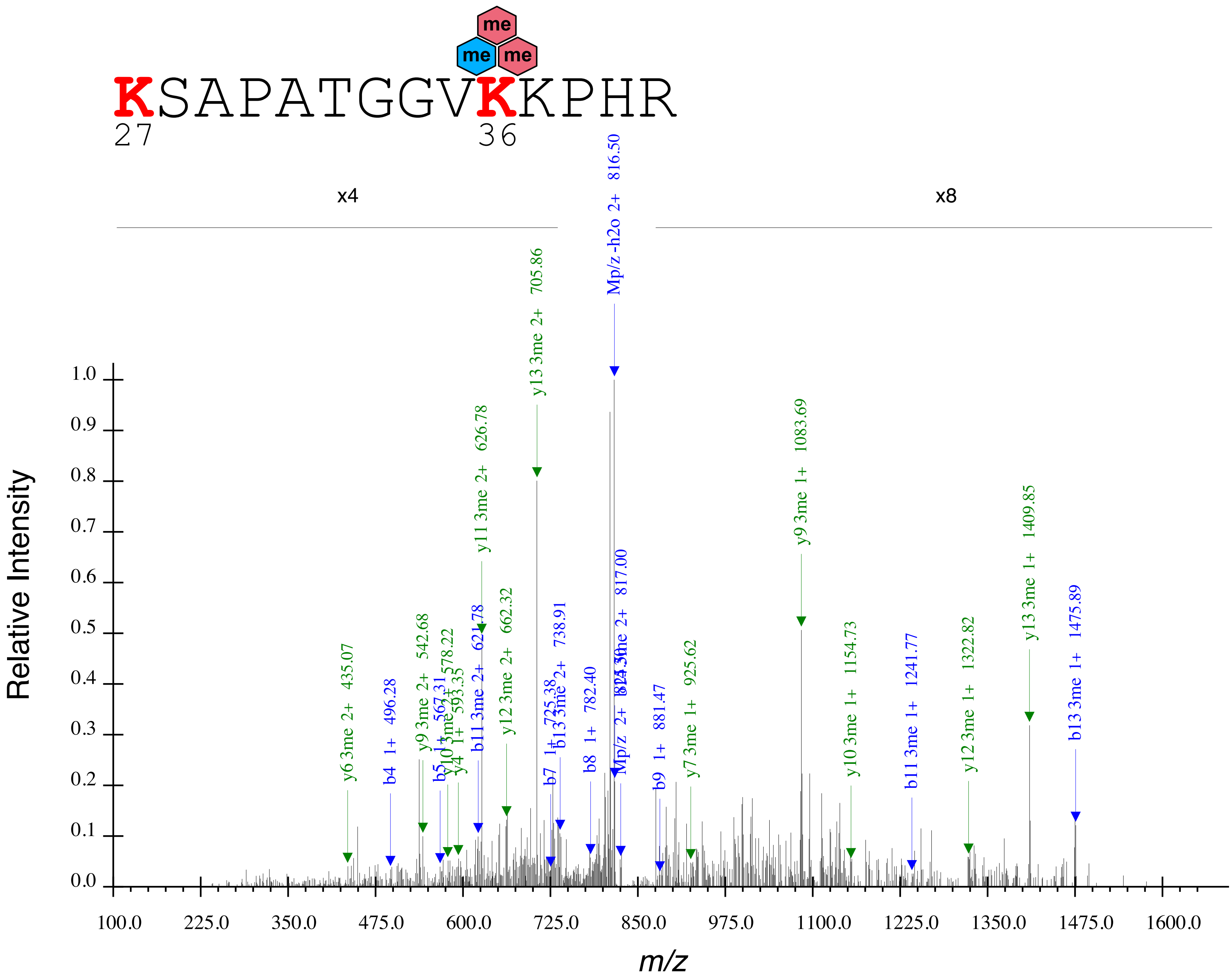

Figure S19 | Annotated CID tandem mass spectrum for H3K27unK36me3(CH_3,2CD_3), z = 2, Mp/z = 825.50. N-terminus, K27 and K37 have been propionylated (+56.0627 Da).

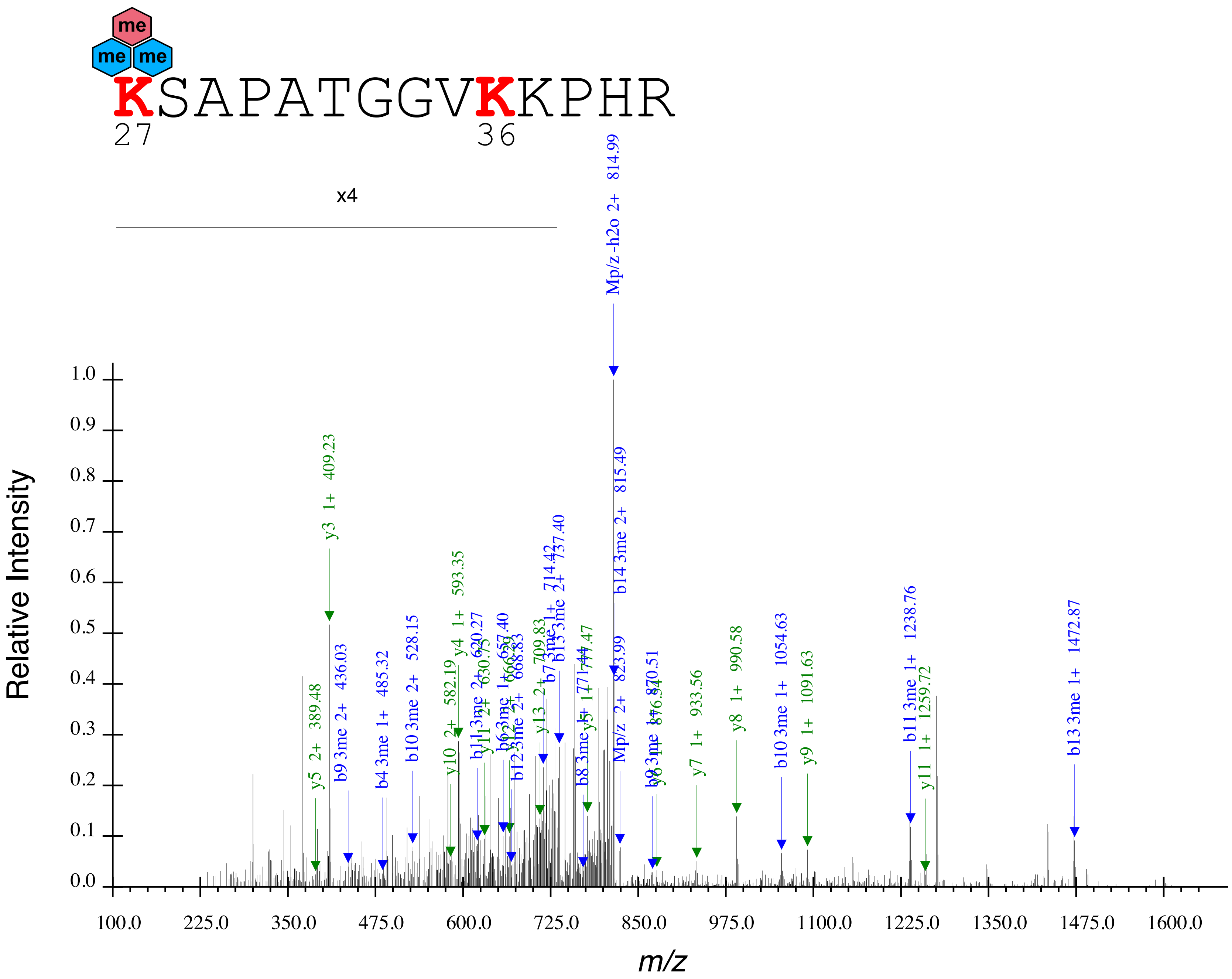

Figure S20 | Annotated CID tandem mass spectrum for H3K27me3(2CH_3,CD_3)K36un, z = 2, Mp/z = 823.99. N-terminus, K36 and K37 have been propionylated (+56.0627 Da).

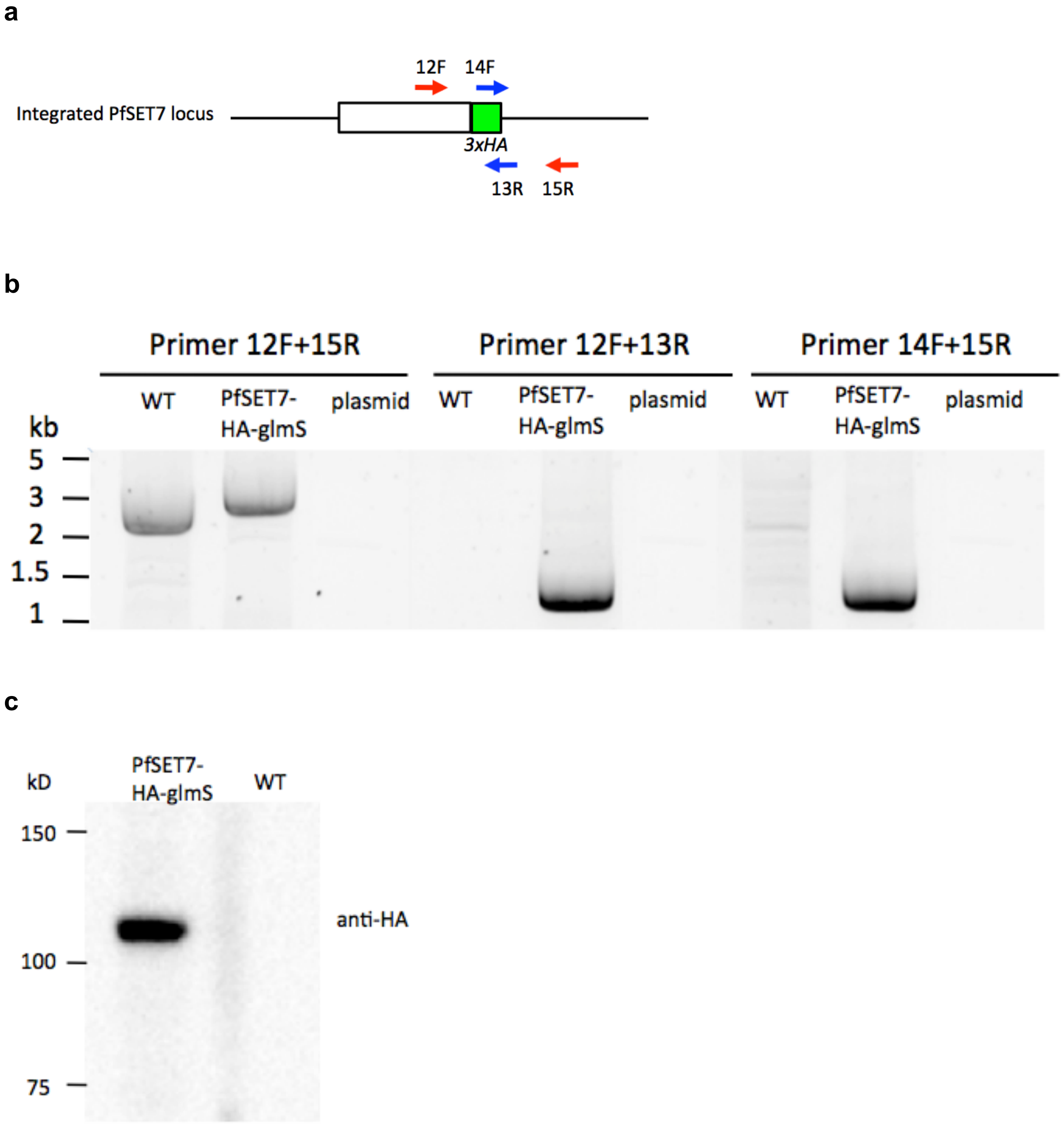

Figure S21 I **Confirmation of PfSET7-HA-glmS integration.** (a) Schematic of the PCR verification of PfSET7-HA-glmS integration. White rectangles represent the wild PfSET7 locus and green box indicate the 3xHA tag. Red arrows represent the primers used to identify the upstream and downstream of homologous region. Blue arrows represent the primers located in HA tag sequence. (b) Agarose gel electrophoresis of the upstream, downstream and insertion confirmation PCRs. Left panel: Either wild-type (2248 bp) or the integrated allele (2615 bp) are visualized after amplification with 12F+15R. Middle and right panel: note the presence of integration after amplification with 12F+13R (1230 bp) and 14F+15R (1247 bp). (c) Western blot of PfSET7 in transgenic PfSET7-HA-glmS line and wild-type parasite lines. The anti-HA antibody recognizes a band of >100 kDa (PfSET7-HA predicted MW: 98 kDa).

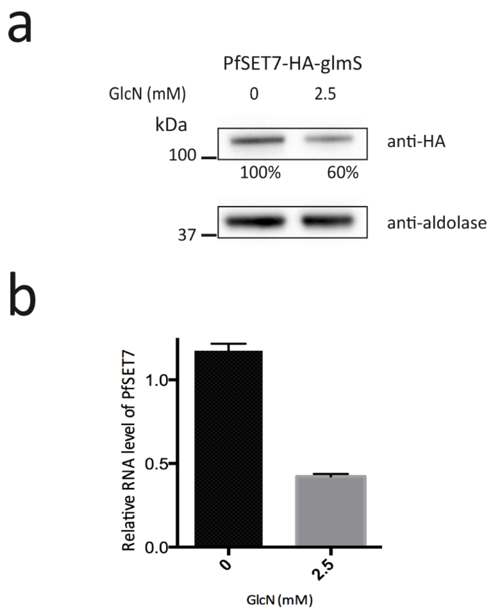

Figure S22 I **Generation of a PfSET7 knock-down line after 2 generations of glucosamine treatment**. (a) Western blot analysis of treated PfSET7-HA-glmS parasites shows 40% decrease in protein levels compared to the control. The PfSET7 expression was normalized to the protein aldolase. The blot image presented has been cropped to the area used for densitometry. (b) Quantitative PCR analysis showing 60% reduction of PfSET7 mRNA levels in treated parasites. Seryl-tRNA synthetase (PF07_0073) was used as an endogenous control. The ΔCT for the PfSET7 gene was determined by subtracting the PfSET7 gene CT value from the seryl-tRNA synthetase gene CT value. Copy number was then converted using the formula (copy number = $2^{\Delta Ct}$). Data are presented as mean ± SD of two independent experiments.

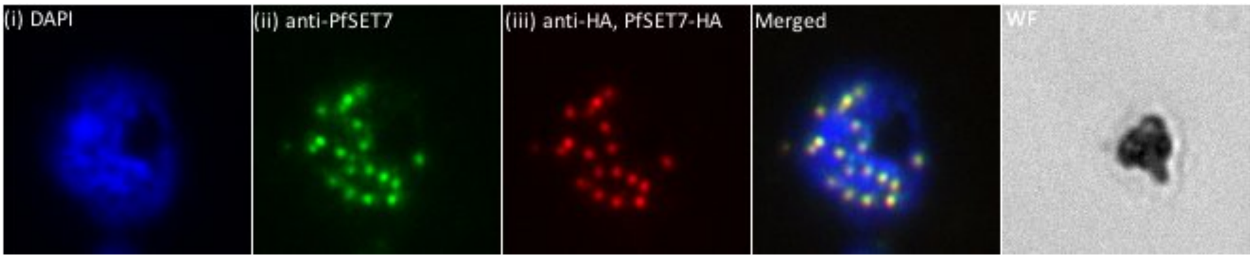

Figure S23 I **Comparison of anti-PfSET7 and anti-HA antibodies in the PfSET7-HA-glmS parasite line.** Both anti-PfSET7 and anti-HA produced a strong punctate staining dots that are consistent with each other. They are equivalent in detecting PfSET7.

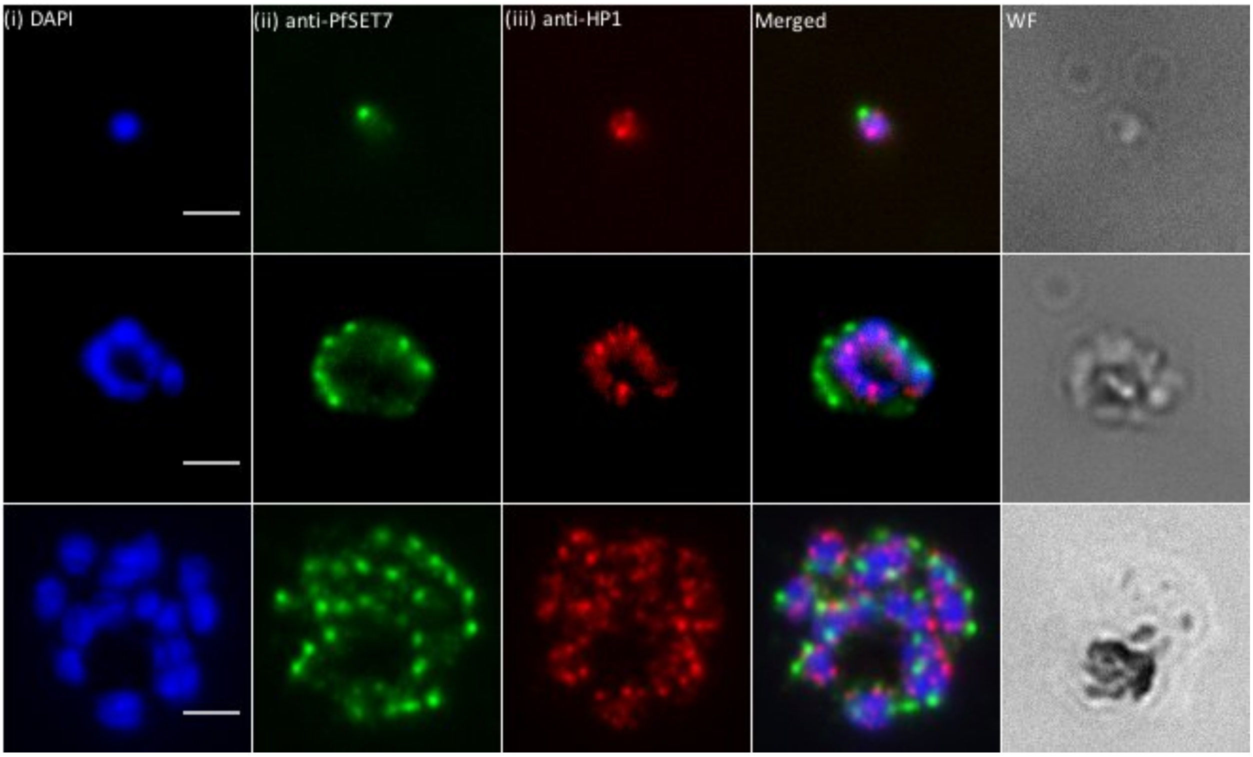

Figure S24 I **Cellular localization of PfSET7 at different blood cell stages.** IF of *P.falciparum* erythrocytic stage rings, trophozoites and schizonts using anti-PfSET7 combined with the nuclear periphery marker PfHP1. Throughout the three asexual blood stages, PfSET7 shows a punctate pattern adjacent to nucleus and distant from nuclear periphery. Scale bar is 2 µm.

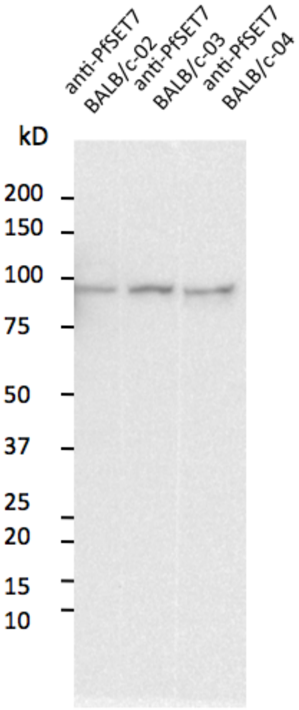

Figure S25 I **Western blot of parasite lysates using the native PfSET7 antibody**.
The anti-PfSET7 antibody from three separate animals all recognize a single band representing the 94 kDa native PfSET7 protein. Identical lanes from a single membrane were cut, probed with the three antibodies individually and then imaged together as oriented on the original membrane.
